# Functional role of a tethered domain as a naturally fused cognate partner is demonstrated in a three-domain copper nitrite reductase

**DOI:** 10.1107/S2052252526004549

**Published:** 2026-06-15

**Authors:** Nopphon Petchyam, Allegra Mbouku, Robert R. Eady, S. Samar Hasnain, Svetlana V. Antonyuk

**Affiliations:** a Molecular Biophysics Group, Life Sciences Building, Institute of Systems, Molecular and Integrative Biology, Faculty of Health and Life Sciences University of Liverpool LiverpoolL69 7ZB United Kingdom; b Center for Advanced Therapeutics, Institute of Molecular Biosciences, Mahidol University, Nakhon Pathom, 73170, Thailand; Chinese Academy of Sciences, China

**Keywords:** protein–protein interactions, denitrification, catalysis, proton transfer, electron transfer, metalloenzymes, enzyme mechanisms, multi-protein complexes

## Abstract

Copper-containing nitrite reductases (CuNiRs) catalyse the reduction of nitrite to nitric oxide and are a key enzyme in the anaerobic ammonium oxidation and denitrification steps of the nitro­gen cycle. The recent recognition of the widespread distribution of three-domain CuNiRs where cognate redox partners are fused to the core NiR enzyme offered the possibility of studying coordinated events (*e.g.* proton-coupled electron transfer) in a conformationally stable donor–acceptor complex. The C-terminal cytochrome *c* tethered domain of the CuNiR from *Ralstonia pickettii* (*Rp*NiR) has been well studied. Reverse engineering of *Rp*NiR undertaken to remove the cognate partner domain showed that the presence of the additional domain resulted in significant differences in the apparent *K*_m_ for nitrite and the reduction potentials of the Cu centres when compared with the core enzyme. The oxidation state of the haem centre and the position of the tethering linker have also been shown to control access of substrate to the active site. A key feature of this control is a conserved tyrosine residue (Tyr323 in *Rp*NiR) located in the tethering linker between the fused domain and the core enzyme. To gain insight into this control, we have undertaken targeted mutations of *Rp*NiR to probe the so-called primary proton channel and perturb putative electron transfer routes from the haem to the ‘gatekeeper’ Tyr323 and to the T1Cu centre. The resolution of our crystallographic data to better than 1.2 Å enabled us to apply unrestrained *SHELXL* refinement of the structures. Our data provide a significant advance in our understanding of catalysis and modulation of electron transfer in these tethered systems, with wider implications for these fundamental processes in other protein complexes.

## Abstract 2

The combination of targeted mutations of a C-terminal cytochrome *c* tethered copper-containing nitrite reductase with functional studies and atomic resolution structures suggests complex roles of the tethered domain rather than simple fusion of a redox partner.

## 1. Introduction

Electron transfer (ET) reactions underpin a wide variety of fundamental life-sustaining processes. Key to these reactions is the formation of protein–protein ET complexes. These complexes are transient, which makes their structural elucidation particularly challenging. Tethered complexes, where a potential electron-donating protein is naturally fused as an additional domain to an enzyme, such as three-domain CuNiRs, have been regarded as tractable self-contained systems that can model encounter complexes. Studies of these tethered systems, like those of chemically cross-linked complexes, may provide insight into some of the key ET reactions in encounter complexes.

Denitrification is the process in which some micro-organisms shift from using di­oxy­gen to couple respiratory ATP synthesis to the reduction of nitrate and nitrite, *via* the gaseous nitrogen oxide intermediates NO and N_2_O, to N_2_. This pathway in the global nitro­gen cycle has important economic and agronomic costs arising from losses of soil nitrogen available for crop growth, and environmental impact, being the major source of the greenhouse gas N_2_O. Nitrite reductases perform the first committed step of denitrification (Zumft, 1997 ▸), since they generate a gaseous product from the reaction
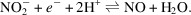
The two-domain Cu-containing nitrite reductases (CuNiR, encoded by *nirK*) have been extensively studied and shown to be trimeric, with each monomer having two domains with a characteristic β-sandwich motif. Each monomer contains a type 1 Cu (T1Cu) electron-accepting centre and a type 2 Cu (T2Cu) catalytic site at the interface between two monomers. The sources of electrons for periplasmic two-domain CuNiRs are either azurin/pseudoazurin or small mono-haem cytochrome *c* (cyt *c*_549,_ cyt *c*_550_, cyt *c*_551_) depending on the organism. These electron donors obtain their electrons from the cytochrome *bc*_1_ complex of the respiratory chain. The activity of CuNiRs requires the formation of a donor–acceptor electron transfer complex as an essential step in catalysis. Such functional complexes are transiently formed between specific proteins, which makes the structural characterization of these complexes challenging. Despite extensive efforts to obtain structures of these catalytically important complexes, only two X-ray structures have been reported, the *Axg*NiR–cyt *c*_551_ complex at 1.7 Å resolution and the heterologous *Ax*NiR–pseudoazurin at 3.0 Å resolution, and the gadolinium-labelled NMR structure of *Af*NiR–pseudoazurin (Nojiri *et al.*, 2009 ▸; Vlasie *et al.*, 2008 ▸; Nojiri, 2016 ▸). These have suggested different electron transfer paths from the redox partner to the core CuNiR domain.

The key in protein–protein interactions lies in the three-dimensional structure that defines the dynamics and specificity of the interacting protein pairs (Berggård *et al.*, 2007 ▸). Despite the high abundance of protein–protein interactions and protein complexes, our understanding of the molecular function of these interactions, including those in protein complexes, is very limited by the scarcity of structural information. In many metalloenzymes such as cytochrome *c* oxidase, hydrogenases, nitro­genases and nitrite reductases, protein–protein interactions are an intrinsic part of catalysis.

The discovery of new classes of CuNiRs where cognate redox partners are fused to the CuNiRs (Ellis *et al.*, 2007 ▸; Han *et al.*, 2012 ▸; Antonyuk *et al.*, 2013 ▸; Nojiri *et al.*, 2007 ▸; Opperman *et al.*, 2019 ▸; Yamaguchi *et al.*, 2004 ▸) may confer an advantage in highly reducing environments where nitrite concentrations are low. This arises from their resistance to reductive inactivation, which prototypic two-domain CuNiRs are susceptible to (Hough *et al.*, 2005 ▸; Strange *et al.*, 1999 ▸; Wijma *et al.*, 2006 ▸; Wijma *et al.*, 2007 ▸). These variants are widespread, and recent data suggest that at least ∼30% of *nirK*-dependent denitrifiers have extended CuNiRs with additional tethered domains such as a cupredoxin or cytochrome *c* (cyt *c*) at either the C or N terminus (Ellis *et al.*, 2007 ▸). Surprisingly, despite the electron-donor proteins fused to CuNiR, which would be expected to minimize the conformational search and lead to an enhancement of inter-protein ET, these tethered enzymes have an order of magnitude lower enzymatic activity, questioning the functional role of the tethered domain.

The only characterized three-domain CuNiRs with a C-terminal cyt *c* extension are those from *Ralstonia pickettii* (*Rp*NiR) (Antonyuk *et al.*, 2013 ▸) and *Pseudoalteromonas haloplanktis* TAC125 (*Ph*NiR) (Tsuda *et al.*, 2013 ▸). Crystallographic studies show that these enzymes retain the trimeric prototypic CuNiR structure with the catalytic core containing two types of copper centres: a T1Cu site coordinated by conserved Cys-Met-His_2_ and a catalytic T2Cu site located at the interface of two subunits and coordinated by three His residues. The two proton-providing residues Asp (Asp_CAT_) and histidine (His_CAT_) in the catalytic pocket characteristic of two-domain CuNiRs are retained in these tethered CuNiRs. In *Rp*NiR, the catalytic core is connected *via* a 36-residue tethering linker to the cyt *c* domain. The crystal structures of both enzymes show that the haem centre of the tethered cyt *c* domain is positioned above the T1Cu site of the core, with an extensive water network at the domain interface. The 10.6 Å separation of the haem and the T1Cu of the adjacent subunit is a distance that would facilitate ET from haem to the T1Cu site. However, in *Rp*NiR this reaction does not occur to any significant extent in the absence of nitrite despite the compatible reduction potentials of the T1Cu and the haem centres, which is attributed to the gatekeeper role of Tyr323 (Hedison *et al.*, 2019 ▸).

In the two-domain prototypic CuNiRs, two potential proton channels from bulk solvent to the T2Cu centre have been identified. However, despite the structural similarity of the core enzyme, in *Rp*NiR the putative hydro­phobic substrate access channel lacks H_2_O molecules and is blocked by a conserved Tyr residue in the domain linker (Tyr323 in *Rp*NiR and Tyr313 in *Ph*NiR). Tyr323 interacts with Asp_CAT_ and also a nearby water (W2) (Antonyuk *et al.*, 2013 ▸). The non-functional water channel between two monomers seen in prototypic two-domain CuNiRs predominates in both *Rp*NiR and *Ph*NiR. The linker that carries Tyr323 has been suggested to have a role in modulating the enzymatic activity by offering conformational flexibility at the interface between the CuNiR core and the cyt *c* domain (Hedison *et al.*, 2019 ▸). The unexpected conformational flexibility of the linker, which assumes a β-sheet in wt*Rp*NiR and a random coil in the isolated core, allowed rationalization of a low-resolution small-angle X-ray scattering (SAXS) structural model of the native enzyme in solution that placed the haem-to-T1Cu distance at ∼40 Å (Han *et al.*, 2012 ▸). This extended conformation, together with the unfavourable driving force for ET, in the case of *Rp*NiR (haem +290 mV, T1Cu +266 mV, T2Cu +255 mV) offers a neat explanation for the slow rates of ET observed in solution. Electron donation *in vivo* to this class of NiRs is likely to involve both periplasmic and membrane-bound proteins, since in the extended solution conformation the T1Cu centre of the core enzyme becomes exposed, and in the case of *Ph*NiR the cognate cyt *c*_549_ has been shown to be an effective electron donor (Nojiri *et al.*, 2007 ▸). However, given the diversity of microbial electron transfer chains, the linked haem domain could potentially accept electrons from complex III (*cbb*_3_) or other membrane-associated haem *c* donors depending on the organism and the environmental conditions (Hopper *et al.*, 2013 ▸).

Biophysical measurements, together with crystal soaking studies, have shown that oxidized *Rp*NiR does not bind nitrite (Han *et al.*, 2012 ▸), in contrast to prototypic two-domain CuNiRs. However, reductive titration studies revealed that reduction of the haem enabled nitrite to bind tightly to the T2Cu, inducing inter-Cu ET. This lack of ET to the T2Cu in the absence of nitrite protects *Rp*NiR from reductive inactivation, a consequence of the release of the coordinated water from the T2Cu site on reduction (Hough *et al.*, 2005 ▸; Strange *et al.*, 1999 ▸; Wijma *et al.*, 2006 ▸; Wijma *et al.*, 2007 ▸). These observations suggest that the reduction of the haem may stabilize the compact structure, as seen in the crystal structure. The role of Tyr323 (conserved in all sequences of cyt *c*-extended CuNiRs) in modulating substrate access was first investigated by crystallographic comparison of native *Rp*NiR exposed to NO and the structures of the NO- and nitrite-bound Asp_CAT_Asn variant (Dong *et al.*, 2018 ▸). Exposure of crystals of *Rp*NiR to NO resulted in the rotation of Tyr323 with the accompanying hydrogen-bonded H_2_O molecule moving away from Asp_CAT_ and the Ser315–Ser321 loop adopting an open conformation, allowing NO to bind to the T2Cu. Similar treatment of the Asp_CAT_Asn variant also allowed nitrite to bind, enabling the first structure of a nitrite-bound enzyme of this class of CuNiRs to be determined (Dong *et al.*, 2018 ▸). Subsequently, the Tyr323Ala/Phe/Glu variants were generated; all were active despite the linker loop being in the locked-down position (Hedison *et al.*, 2019 ▸).

We report near-atomic and high-resolution crystal structures of di­thio­nite-reduced *Rp*NiR for the first time, together with variants of residues (in some cases also NO-bound species) implicated in different aspects of catalysis. These targeted the communication between the cyt *c* domain and the core enzyme (Ser321Met); the proton-gated electron transfer between T1Cu to T2Cu (Met148Leu); and the primary proton channel mutants (Gln262Asn and Phe295Leu) that disturb the enzymatic activity of the enzyme. The resolution of these four *Rp*NiR structures is sufficiently high (<1.2 Å) to allow us to perform unrestrained *SHELXL* refinement together with that of the 1 Å wild-type *Rp*NiR structure in the resting state (3ziy). Comparing the protonation states of catalytic residues between oxidized and reduced *Rp*NiR highlights the reason why exposure to reductants in the absence of substrate does not inactivate *Rp*NiR. In addition, our observation of a new conformation of Tyr323 and the linker loop in the Phe295Leu *Rp*NiR provides insight into the mechanism of Tyr activation in tethered CuNiRs. This rearrangement of Tyr323 results in more than doubling of the activity of the Phe295Leu mutant compared with the wt*Rp*NiR. The wild-type enzyme and all the mutants show similar bell-shaped pH profiles with an activity maximum around pH 5.5, similar to prototypic two-domain CuNiRs. Our data demonstrate that the interaction of the tethered domain with the core enzyme is functionally complex, rather than a simple fusion of a protein and its cognate redox partner aimed at restricting conformational flexibility to provide more effective electron transfer.

## 2. Materials and methods

### 2.1. Protein expression, and purification of *Rp*NiR mutants

The plasmid encoding sequences of *Rp*NiR mutants Ser321Met, Met148Leu, Phe295Leu and Gln262Asn were custom-synthesized (Genscript). The culture and expression of the *Rp*NiR variants were as described previously (Antonyuk *et al.*, 2013 ▸; Han *et al.*, 2012 ▸). The bacterial cells were re-suspended in buffer A (20 m*M* Tris–HCl, pH 8.4) supplemented with Protease Inhibitor Cocktail (Roche), lysed by sonication and cell debris removed by centrifugation. The clear supernatant was loaded on a DEAE column (Sigma), pre-equilibrated with buffer A. The column was washed with buffer containing 20 m*M* NaCl, and *Rp*NiR eluted by increasing the NaCl concentration to 50 m*M*. The red fraction containing *Rp*NiR was concentrated by ultrafiltration (Amicon centrifugal filter, 30 kDa cutoff) and applied onto a Superdex 200 16/60 gel filtration column equilibrated with 20 m*M* Tris–HCl, pH 7.5, 200 m*M* NaCl. Two coloured fractions with elution volumes corresponding to the monomeric and trimeric *Rp*NiR were obtained. The monomer fraction was dialyzed for 15 h against 20 m*M* Tris–HCl, pH 7.5, containing 200 m*M* NaCl and 0.1 m*M* CuSO_4,_ an established procedure for re-incorporating Cu into the T2Cu site of *Rp*NiR (Han *et al.*, 2012 ▸). The enzyme was then re-chromatographed on Superdex 200 to separate the trimer from the monomer/trimer mixture. *Rp*NiR trimer fractions were pooled, concentrated by ultrafiltration and stored at −80°C until used.

### 2.2. Crystallization, data collection and structure determination

*Rp*NiR mutants (4 mg ml^−1^ or 7 mg ml^−1^) were crystallized by the hanging-drop method by mixing 2 µl of the protein solution with 1 µl of reservoir solution containing 100 m*M* Bis-Tris propane pH 7.7, 200 m*M* sodium citrate and 22% PEG 3350 at 4°C. Crystals usually appeared in 1–2 weeks, with the *I*2_1_3 form growing faster than the *H*3 form. The soaking solutions for reduced and NO-treated crystals were saturated with N_2_ gas before the experiments. To make an NO-saturated solution, 5 ml of oxygen-free cryoprotectant solution (crystallization solution supplemented with 10–15% of glycerol) was transferred to a capped vial, and 20 ml of NO gas was injected into the solution. Crystals of Gln262Asn were soaked in NO-saturated preservative solution at 4°C. For Phe295Leu, serial soaking was performed by the transfer into 1/4, 1/2 and no dilution of NO soaking solution. Crystals of reduced *Rp*NiR were obtained by including sodium di­thio­nite (5 m*M*) in the cryoprotectant. Following this treatment, crystals were flash-frozen in liquid nitro­gen. Diffraction data were collected from single crystals at 100 K on the I24, I03, I04 and I04-1 beamlines at the Diamond Light Source (Harwell, UK). The crystallographic data set for the Met148Leu mutant was collected on the PROXIMA-1 beamline at the Synchrotron SOLEIL (France). The total X-ray dose for each structure was calculated using *RADDOSE-3D* (Zeldin *et al.*, 2013 ▸). The data were integrated using *DIALS* (Winter *et al.*, 2018 ▸) or *iMOSFLM* (Battye *et al.*, 2011 ▸) and scaled using *Aimless* (Evans & Murshudov, 2013 ▸) in the same origin as wt*Rp*NiR (PDB ID: 3ziy) for all mutants that crystallized in the *H*3 space group. Their structures were refined with *Refmac5* (Murshudov *et al.*, 2011 ▸) using wt*Rp*NiR (PDB ID: 3ziy) as the starting model. For Ser321Met, which crystallized in the *I*2_1_3 space group, the same starting model was used for molecular replacement with *MOLREP* (Vagin & Teplyakov, 2010 ▸) software as a part of the *CCP4* program suite. Restrained anisotropic refinement was performed with *Refmac5* (Murshudov *et al.*, 2011 ▸) for atomic resolution (above 1.2 Å) structures and isotropic refinement for those with medium resolution. The structures were manually adjusted using *COOT* (Emsley & Cowtan, 2004 ▸), water molecules and ligands were gradually added, and at the last stage of the refinement riding hydrogen atoms were added. The structures were validated using the PDB validation server (Gore *et al.*, 2017 ▸). For atomic resolution, the structures of reduced wt*Rp*NiR, as-isolated Phe295Leu *Rp*NiR, NO-soaked Phe295Leu *Rp*NiR and NO-soaked Gln262Asn *Rp*NiR were further refined by *SHELXL* (Sheldrick, 2008 ▸). The oxidized structure of wt*Rp*NiR (3ziy) was also re-refined by *SHELXL*. The procedure included refinement of occupancies of double conformations, anisotropic *B* factors and T2Cu ligands. For atomic resolution structures, one cycle of unrestrained block-matrix least-squares refinement was performed as the final step. The positions of hydrogen atoms were investigated by analysis of hydrogen omit *F*_o_ − *F*_c_ maps. As some of the catalytic residues did not show any hydrogen atoms, we measured the bond lengths and angles (and their associated e.s.d. values) to suggest the protonation state of catalytically important residues.

### 2.3. Specific activity assay of *Rp*NiR mutants

The activity of *Rp*NiR mutants (Ser321Met, Met148Leu, Phe295Leu and Gln262Asn) was measured using NADH/phenazine metho­sulfate as a reductant. This has been shown to be a highly effective reductant for CuNiR (Kobayashi *et al.*, 1999 ▸). The spectrophotometric assay described here is a modification allowing the continuous measurement of activity, rather than periodic sampling for product (NO) analysis by mass spectrometry. The reaction was performed under nitro­gen gas (N_2_) in a Suba-sealed quartz cuvette to maintain anaerobic conditions. The assay mixture contained 50 m*M* MES (pH 5.5), 1 m*M* NaNO_2_, 5 µ*M* phenazine metho­sulfate (PMS) and 4 m*M* NADH as the electron donor. The reaction was initiated by the addition of 1.7 n*MRp*NiR. Nitrite reductase activity was monitored in real time by measuring the decrease in absorbance of NADH at 340 nm corresponding to the oxidation of NADH with a Cary 3500 UV–Vis Spectrophotometer (Agilent) using an extinction coefficient of 6220 *M*^−1^ cm^−1^. The rate of NADH consumption was calculated from the initial slope of the decrease in absorbance. Enzyme activity is expressed as half the amount of NADH consumed, since each NADH molecule donates two electrons, while the reduction of one nitrite molecule requires only one electron:
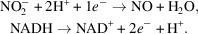


## 3. Results and discussion

### 3.1. Protonation status of catalytic residues of *Rp*NiR in the resting oxidized and di­thio­nite-reduced states revealed by unrestrained *SHELXL* refinement

The structure of reduced wt*Rp*NiR was solved in the space group *H*3 at 1.17 Å resolution, which, together with our 1.01 Å structure of the oxidized resting state (Ellis *et al.*, 2007 ▸), enabled unrestrained refinement implemented in *SHELXL* (Sheldrick, 2008 ▸, 2015 ▸) for both redox states. Unrestrained *SHELXL* refinement is a robust approach for determining unbiased atomic positions. The method requires atomic resolution diffraction data to perform full-matrix least-squares inversion, which in turn yields gold-standard error estimates for the refined parameters. The precise atomic positions can help in assigning the protonation states of catalytic residues, reflected in characteristic bond lengths and angles (Rose *et al.*, 2026 ▸). To ensure statistical rigour, we employed *Z*-scores (*N*σ) to quantify the confidence level of each designation (Table S1). The *SHELXL* refinement enabled details of the proton delivery and substrate access channels, the Tyr323 substrate-binding switch and the protonation status of important residues in the catalytic core to be determined and compared.

**Figure 1:**
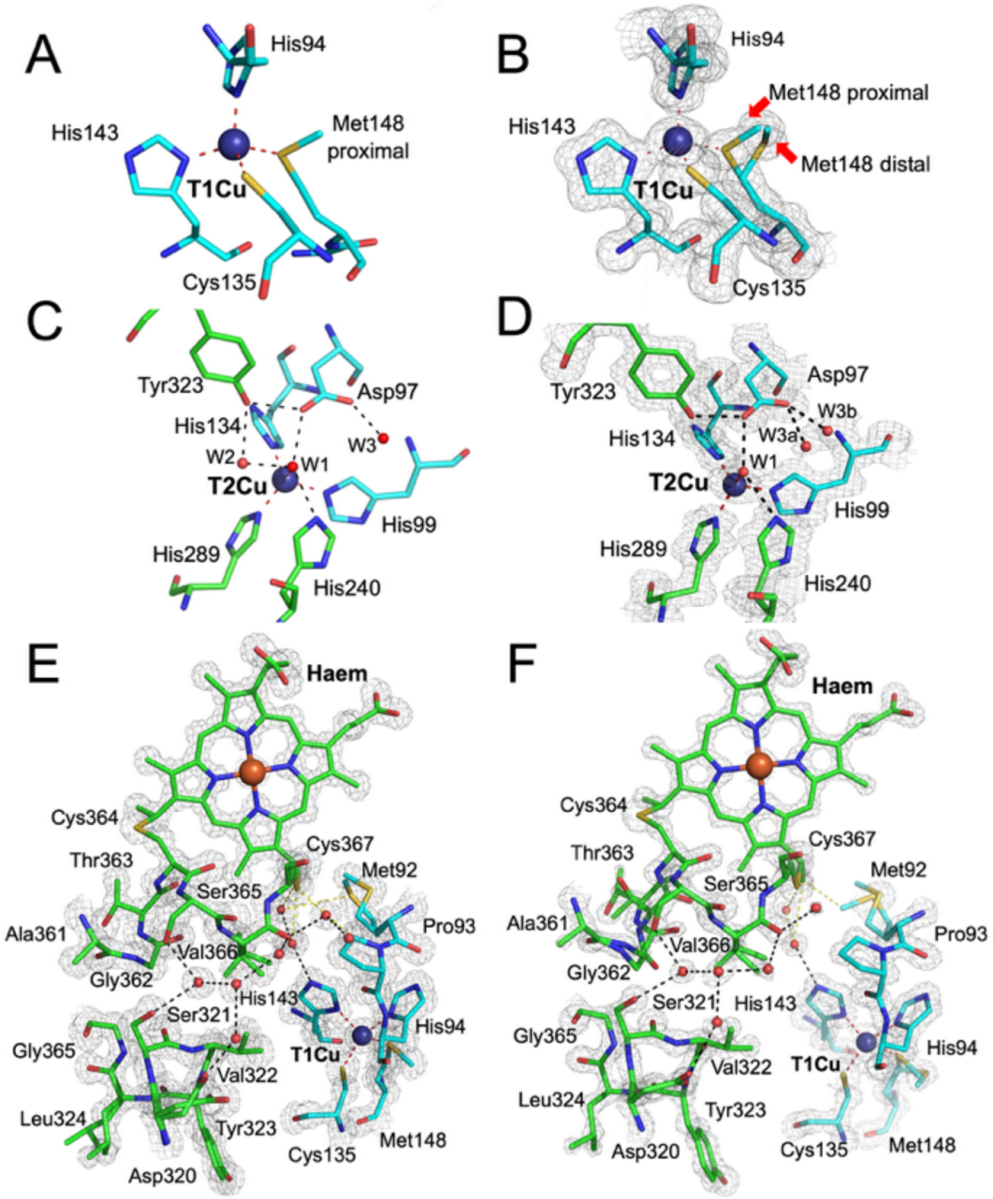
Comparison of T1Cu and T2Cu sites and the interface of the cyt *c* domain in wt*Rp*NiR, as-isolated, oxidized and reduced. (A) T1Cu site of oxidized *Rp*NiR (3ziy) shows an ideal tetrahedral geometry. (B) In contrast, in the reduced enzyme, Met148 adopts two conformations, with T1Cu to S^δ^ distances of 2.67 and 4.4 Å. (C) T2Cu site of as-isolated wt*Rp*NiR (3ziy). (D) T2Cu site of reduced wt*Rp*NiR with Cu bound to a single water, W1. Loss of W2 likely weakens the anchoring of the gatekeeper residue Tyr323. (E) Conformation of residues involved in electron transfer at the interface between the cyt *c* domain and the core domain in as-isolated wt*Rp*NiR (3ziy). (F) The inter-domain interface in reduced wt*Rp*NiR reveals the flexibility of Gly362 and Thr363, present in two conformations. The residues from neighbouring molecules are coloured green and blue. The 2*F*_o_ − *F*_c_ electron-density map is shown as a grey mesh at the 1σ level. The red spheres represent the water molecules and the deep blue spheres the Cu ions. Important hydrogen bonds are shown as black dashed lines; yellow dotted lines represent through-bonded interactions and red dashed lines are metal coordinating bonds.

In the *SHELXL* re-refined structure of oxidized wt*Rp*NiR (3ziy**)**, the water ligand (W1) is bound to the catalytic T2Cu site at 2.067 (7) Å (Fig. 1 ▸, Table S2). A second water molecule (W2) is hydrogen-bonded to W1 (2.4 Å) and the OH group of Tyr323 (2.8 Å), and is positioned 2.6 Å from T2Cu. (Fig. 1 ▸, Fig. S6). T2Cu ligands have a distorted tetrahedral geometry. By analogy with prototypic CuNiRs, Asp97 and His240 form the catalytic unit with T2Cu and its water ligand (W1). The Asp_CAT_ residue (Asp97) is present in a single conformation (Fig. 1 ▸), and analysis of the electron density and the geometry of the carboxyl group of this residue (Fig. 1 ▸, Fig. S8, Table S1) suggests that this residue is protonated [bond lengths 1.282 (9) Å and 1.242 (8) Å] at O^δ1^ and is neutral. The imidazole ring of the catalytically important residue His_CAT_ (His240) is rotated towards W1 forming a strong hydrogen bond at 2.6 Å by the N^ɛ2^ atom, while O^δ1^Thr263 is hydrogen-bonded to N^ɛ1^ at 2.9 Å. This differs from two-domain CuNiRs, where N^δ1^His_CAT_ is hydrogen-bonded to Asp_CAT_ via water (Ellis *et al.*, 2003 ▸; Rose *et al.*, 2021 ▸). In *Rp*NiR the C—N—C angles associated with the N^δ1^ and N^ɛ2^ atoms of His_CAT_ are 108.5 (6)° and 104.5 (6)°, respectively, which correspond to protonated N^δ1^ and non-protonated N^ɛ2^ (Liebschner *et al.*, 2013 ▸; Malinska *et al.*, 2015 ▸) when compared with imidazole groups in the Cambridge Structural Database (CSD) (Allen, 2002 ▸; Groom* et al.*, 2016 ▸). This is further supported by the hydrogen omit map, shown as a red mesh, which displays clear density for the hydrogen atoms at all positions except N^ɛ2^ [Fig. S7(A)].

Compared with the oxidized state, differences in both Cu sites are observed in the 1.17 Å structure of reduced *Rp*NiR [Fig. 1 ▸(B, D)]. The reduced T1Cu site shows two conformations of Met148, which we assign as proximal and distal conformations, The proximal conformation of Met148 (occupancy of 0.6) is in the coordination range of the T1Cu, as seen in oxidized wt*Rp*NiR. In the distal conformation (occupancy of 0.4), the S^γ^ of Met148 is turned away from T1Cu, similar to Met144 in the atomic resolution structure of the as-isolated two-domain *Ax*NiR (Ellis *et al.*, 2003 ▸), which probably indicates that some radiation-induced reduction of the latter has occurred. The distance between Met148 S^δ^ to the T1Cu in the proximal conformation [2.676 (7) Å] is modestly higher than that in the oxidized structure [2.584 (6) Å], and the distance in the distal conformation (4.40 Å) is out of the coordination range of T1Cu. At the catalytic site, only a single W1 is present, and bound to T2Cu with a distance of 2.028 (2) Å, and W3 shows a dual conformation [Fig. 1 ▸(D), Fig. S7]. Some of the hydrogen atoms in the hydrogen omit map observed in the oxidized structure, particularly for Tyr323, are no longer visible in the reduced structure [Fig. S7(B)]. Hydrogen atoms observed at the haem site are shown in Fig. S9.

The geometry of T2Cu is tetrahedral, as in the oxidized state. However, Asp97 is deprotonated and negatively charged [O^δ1^— C^γ^ is 1.270 (9) Å and O^δ2^—C^γ^ is 1.259 (9) Å], while the protonation state of His240 is similar (Fig. S7, Table S1). In summary, two main differences between oxidized and reduced wt*Rp*NiR are the presence of a single water (W1) ligand to the T2Cu of the reduced enzyme, and the deprotonated state of Asp97, which did not affect the conformation of Tyr323. In the resting state, the locked-down position of Tyr323 is stabilized by the interactions with W2 and Asp97 *via* its protonated O^δ2^. Comparing the water network at the interface between the haem *c* and core domains shows that the putative water-mediated ET pathway is retained [Fig. 1 ▸(E, F)]; however, flexibility of the residues Gly362–Thr363 in the cyt *c* domain is observed.

**Figure 2:**
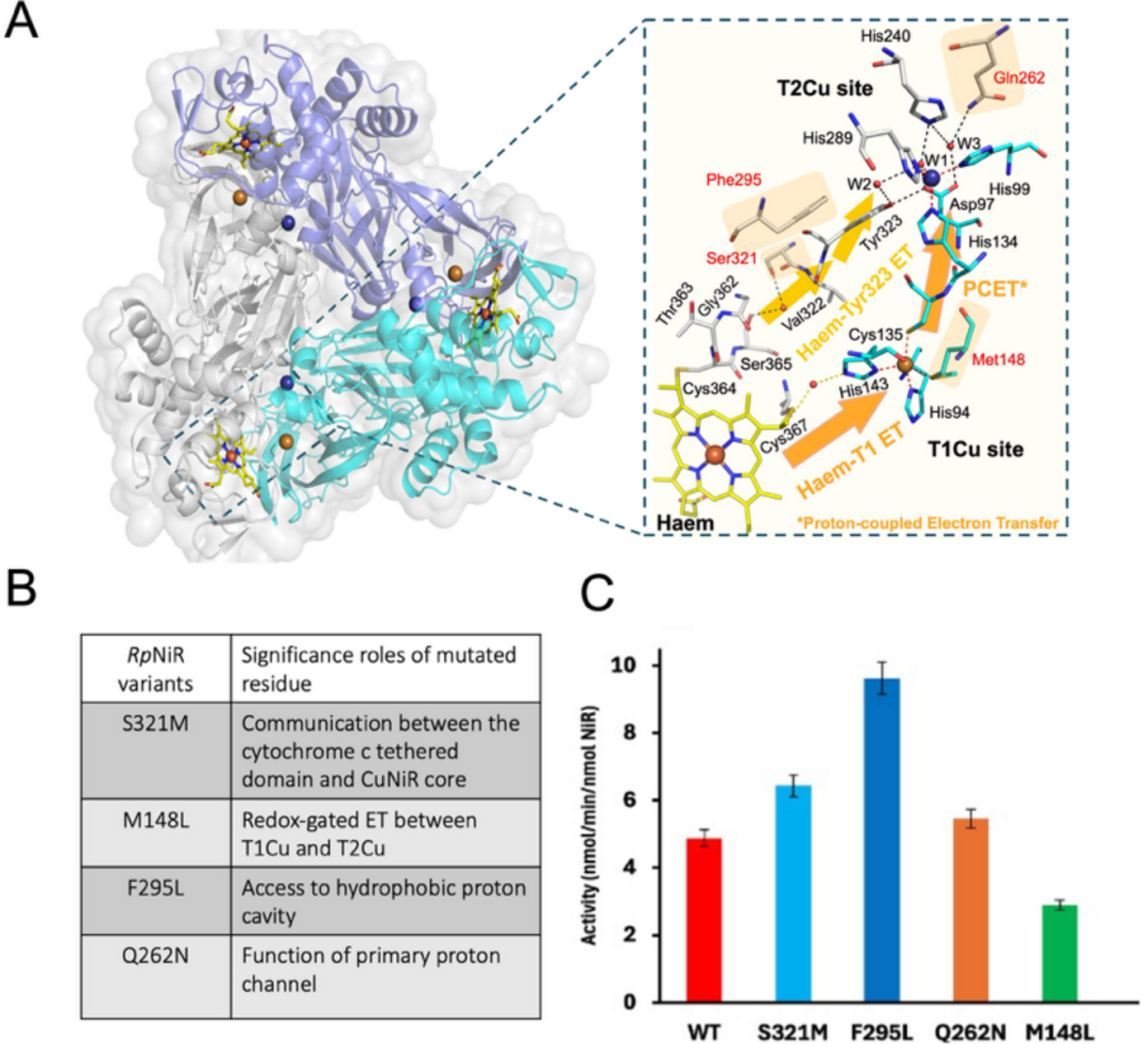
*Rp*NiR mutation sites and specific activity of the mutants. (A) The structure of wt*Rp*NiR is shown as a cartoon with its monomers in different colours: slate, blue and grey. The details of key residues involved in the ET transfer between the cyt *c* domain and the CuNiR core domain are shown. The variants in this study including Ser321Met, Met148Leu, Gln262Asn and Phe295Leu are labelled in red. The water molecules associated with each channel are shown as spheres with the respective colour, otherwise they are shown as red spheres. The copper-coloured spheres are T1Cu, deep blue spheres are T2Cu, the black dashed lines represent hydrogen bonds, yellow dashed lines represent through-bond interactions and red dashed lines represent the interactions involving copper sites. (B) The putative roles of mutated residues are described. (C) The specific activity of *Rp*NiR mutants, including Ser321Met, Met148Leu, Phe295Leu and Gln262Asn, compared with wt*Rp*NiR. Bars represent the mean; error bars indicate the standard deviation of three technical replicates (*n* = 3). pH activity profiles of wt*Rp*NiR and mutants Ser321Met, Met148Leu, Phe295Leu and Gln262Asn are given in Fig. S1.

We also constructed structure-informed point mutations of residues that have been proposed to be involved in different aspects of catalysis that are complex and inter-linked. Mutations were selected to probe ET from the haem group of the tethered cyt to the core domain, inter-Cu ET, improving accessibility to the T2Cu active site pocket and potential disruption of the primary proton channel (Fig. 2 ▸).

### 3.2. Catalytic activity of *Rp*NiR mutants

The specific activity of *Rp*NiR mutants was determined by monitoring NADH oxidation under nitro­gen gas in a Suba-sealed quartz cuvette. The artificial electron donor and mediator were NADH and PMS, respectively. The activities are shown in Fig. 2 ▸(C) with their pH dependence in Fig. S1. All enzymes exhibited optimal activity at pH 5.5. At this pH, the activity of the Phe295Leu mutant more than doubles compared with the wt*Rp*NiR, while the activities of Ser321Met and Gln262Asn are increased by 30% and 10%, respectively. Met148Leu showed a decrease of ∼40%.

In contrast, at pH 6.5, where the activity of other CuNiRs, including tethered NiRs, is generally reported, much larger effects were observed. Phe295Leu showed a fivefold increase in activity, Ser321Met and Gln262Asn a threefold increase, while the Met148Leu variant a 20% decrease (Fig. S1). The bell-shaped activity pH profiles are very similar to the pH dependence profile of *k*_cat_ and the rate of inter-Cu ET of the prototypic *Ax*NiR (Suzuki *et al.*, 2000 ▸). This behaviour is attributed to the involvement of Asp_CAT_ or His_CAT_ in proton delivery in the PCET inter-Cu reaction. The very similar activity pH profiles of *Rp*NiR and *Ax*NiR (Suzuki *et al.*, 1997 ▸; Abraham *et al.*, 1997 ▸; Kobayashi *et al.*, 1999 ▸) indicate that the structural elements that regulate catalysis in CuNiRs are functionally preserved in the haem-tethered enzymes. This differs from the cupredoxin-extended *Hd*NiR, where the pH dependence profile of *k*_cat_ and the rate of inter-Cu ET show no optimum, but a linear decrease over the pH range 4.5 to 7.0 (Eady & Hasnain, 2022 ▸). To better understand these effects in structural terms, we solved the high-resolution crystal structures of these *Rp*NiR variants, as shown in Tables 1 ▸ and 2 ▸.

### 3.3. Mutation of Ser321 in the linker loop increases NiR activity

EPR spectroscopy and crystallographic studies have shown that as-isolated oxidized wt*Rp*NiR does not bind nitrite (Hedison *et al.*, 2019 ▸) but mutation of Tyr323 in the linker loop to Ala, Glu or Phe enabled binding, and these variants have been structurally characterized (Hedison *et al.*, 2019 ▸). These observations indicate that this conserved residue controls access of nitrite to the T2Cu site, functioning as a ‘gatekeeper’ switch for substrate binding in three-domain NiRs (Dong *et al.*, 2018 ▸; Hedison *et al.*, 2019 ▸). Reduction of the haem centre is a pre-requisite for substrate binding with the ‘gatekeeper’ Tyr in an activated conformation, and subsequent inter-Cu ET to the active site is promoted by the increase in potential of the nitrite-bound T2Cu site (Hedison *et al.*, 2019 ▸), as occurs in prototypic CuNiR (Ghosh *et al.*, 2009 ▸). However, there is a paucity of information as to how the *Rp*NiR core in the native enzyme senses the oxidation state of the haem. Some insight has been gained from the structure and properties of the Asp97Asn mutant, which can bind both NO and NO_2_^−^ (Dong *et al.*, 2018 ▸). Both ligand-bound structures showed rotation of Tyr323 together with an accompanying water molecule, and the Ser315–Ser321 loop adopting an open conformation. Also, in the NO-bound structure, Tyr323 moves away from the binding pocket to share a water molecule with Asp320, which connects *via* Ser321–Gly362 to the haem Cys364 (Dong *et al.*, 2018 ▸). Given the connectivity of Tyr323 to the cytochrome domain *via* Ser321–Gly362, we constructed the Ser321Met variant to test its potential for activation of Tyr323 and its impact on enzymatic activity.

**Figure 3:**
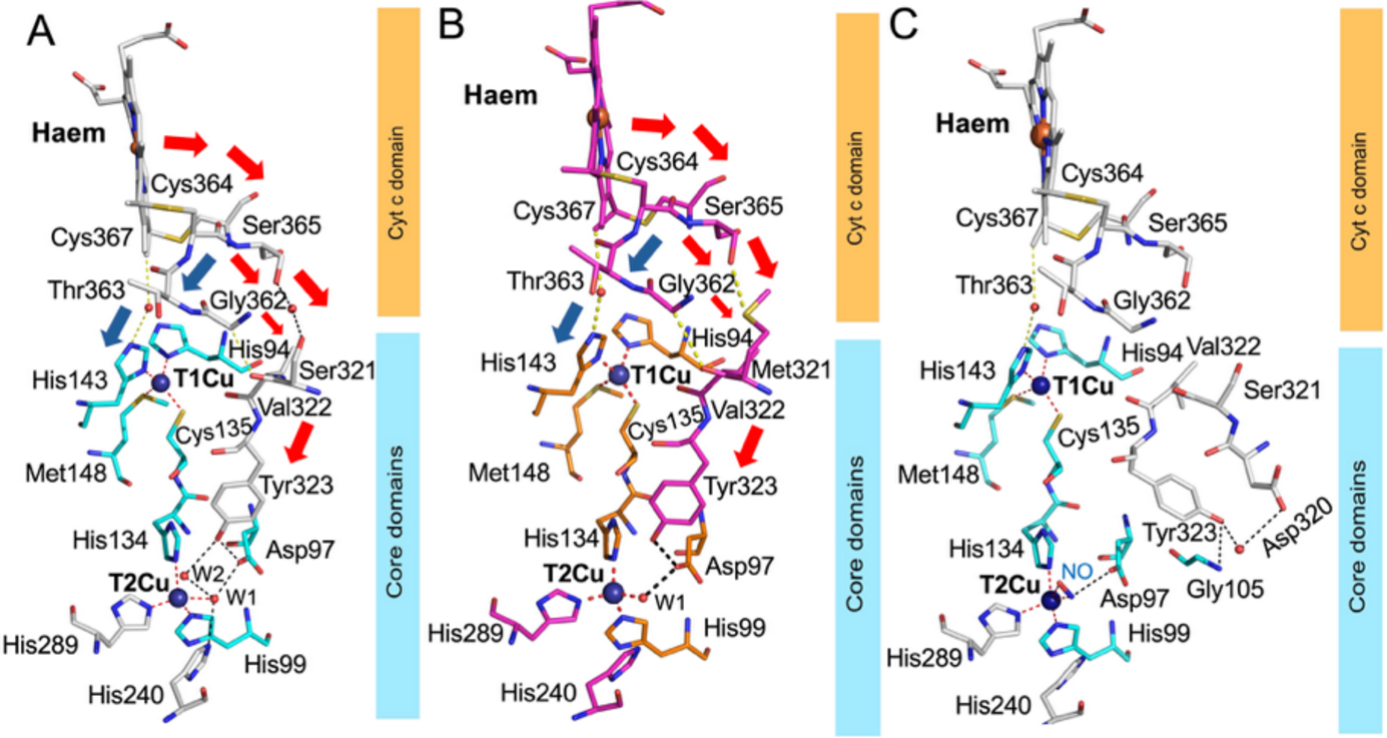
Comparison of the conformation of residues potentially involved in electron transfer at the interface between the tethered cyt *c* domain and the core domain of (A) wt*Rp*NiR, (B) Ser321Met *Rp*NiR and (C) NO-bound wt*Rp*NiR. Potential electron transfer routes from Cys364 to Tyr323 are represented by red arrows, and from Cys367 to His143 by blue arrows. In the Ser321Met mutant, the side chain of Met321 replaces the water that connects the cyt *c* domain to the core domain. Ser321Met is shown in magenta and orange, and wild type is shown in grey and blue. The red spheres are water molecules and deep blue spheres are copper ions. The black dashed lines represent hydrogen bonds, yellow dashed lines through-bonds and red dashed lines the interactions involving copper sites.

The structure of the oxidized Ser321Met variant was determined in the *I*2_1_3 space group at 2.1 Å resolution. Overall, the structure is similar to the wt*Rp*NiR (the overall r.m.s. displacement in C^α^ atoms is 0.15 Å), with the Tyr323 residue in the locked-down position [Fig. 3 ▸(B), Fig. S2]. The side chain of Met321 extends towards Ser365 and replaces the water molecule bridging O^δ1^Ser321 and O carbonyl of Ser365 in wt*Rp*NiR [Fig. 3 ▸(A) and Fig. 1(E)], which positions S^δ^Met321 3.1 Å away from O^δ1^Ser365. Met321 has a single conformation, as clearly seen in the electron density [Fig. S2(A)]. The mutation disturbs the water network at the interface between the cyt *c* and core domains that links Cys364 to the side chain of Asp320 in wt*Rp*NiR [Fig. 3 ▸(A, B)]. While the position of the main chain of the linking loop is unaltered, the neighbouring cupredoxin domain moves 0.4 Å closer to the linker loop in comparison with the wt*Rp*NiR structure. The coordination geometry of the Cu sites in the mutant is unchanged [Fig. S2(C)]. However, the additional water W2, which usually anchors Tyr323 in the catalytic pocket, is absent, creating space for substrate binding at the T2Cu site, which now resembles the typical T2Cu site in prototypic CuNiRs. This substitution, however, resulted in only an ∼25% increase in activity [Fig. 2 ▸(C)] or a threefold increase away from the pH optimum. His_CAT_ is no longer hydrogen-bonded to W1, which forms a strong hydrogen bond at 2.6 Å in the oxidized enzyme, suggesting that substrate access to the catalytic pocket is not a significant rate-limiting factor, consistent with the low apparent *K*_m_ for nitrite (1.6 µ*M*) (Hedison *et al.*, 2019 ▸).

The slow rate of ET from the haem to T1Cu in wt*Rp*NiR observed in laser flash photolysis studies was attributed to rate-limiting searches of conformational space required to optimize electronic coupling between the haem and T1Cu in the compact structure seen in crystallography (Hedison *et al.*, 2019 ▸). We have identified three potential routes for ET from the haem to the T2Cu pocket. The first route *via* the T1Cu is water-mediated and involves Cys367, water and the T1Cu ligand His143 [Fig. 3 ▸(A), blue arrows]. The second and third are branched from Cys364 to the gatekeeper Tyr323, which are haem c > Cys364 > Gly362 > Ser321 > Tyr323, and haem c > Cys364 > Ser365 > water > Ser321 > Tyr323, respectively [Fig. 3 ▸(A)]. The small increase in activity of the Ser321Met variant may arise from a slightly more efficient inter-domain ET, since the second and third ET pathways, which involve the side chain of Met321, are likely to be enhanced by through-bond interaction, in which the distance between C^ɛ^Met321 and O^γ^Ser365 in the cyt *c* domain is shorter (3.16 Å) compared with O^γ^Ser321 and O^γ^Ser365 (3.66 Å) in the wt*Rp*NiR structure [Fig. 3 ▸(A, B)]. These changes may result in the more efficient activation of Tyr323, a potential rate-limiting step in enzyme turnover.

### 3.4. Substitution of the T1Cu Met ligand to Leu suggests haem–T1Cu ET is rate-limiting and confirms the role of the cytochrome *c* domain as a tethered functional donor

In the absence of substrate, the difference between the reduction potentials of the T1Cu and T2Cu sites in prototypic CuNiRs provides only a small, or in some cases an unfavourable, driving force for ET from the T1Cu site (Eady & Hasnain, 2022 ▸). The binding of nitrite to the oxidized catalytic T2Cu results in an increase in reduction potential resulting in the proton-coupled T1Cu–T2Cu ET through an ∼12.6 Å Cys–His bridge connecting the two centres. The effect of the T1Cu axial ligand substitutions on the reduction potential of T1Cu and their effect on activity has been studied in several prototypic CuNiRs. In the case of *Ax*NiR, as expected, the reduction potential of Met144Leu increased by +96 mV to +336 mV, attributed to a weaker axial interaction and a change in the dielectric constant of the T1Cu site (Hough *et al.*, 2005 ▸). The effect of this substitution on activity was dependent on the electron donor. The effect was marginal with the non-physiological donor methyl viologen (∼16% increase) but resulted in ∼70% increase of activity with the putative physiological electron donor azurin (*E*_m_ +305 mV) (Hough *et al.*, 2005 ▸). The much higher increase in enzymatic activity observed with the physiological donor provided strong support to the hypothesis that intermolecular electron transfer (interaction) with the physiological donor (azurin) is the rate-determining step in nitrite reduction by NiR enzymes rather than the intramolecular electron transfer between the T1Cu and T2Cu sites (Hough *et al.*, 2005 ▸).

One of the effects of tethering of the haem domain in *Rp*NiR is the modulation of the reduction potential of the T1Cu site from +266 mV in the native enzyme to +331 mV in the isolated core (Hedison *et al.*, 2019 ▸). This difference did not influence the slow rate of ET from haem (+290 mV) to the T1Cu centre of the core, despite the positive driving force for ET. This was attributed to rate-limiting conformational sampling in the tethered system (*Rp*NiR) or interacting surface rolling in the encounter-complex formation, *i.e.* in the two-component system. Under turnover conditions, the extended conformation of wt*Rp*NiR in solution, as indicated by the SAXS profile, makes it highly likely that the formation of an effective ET conformation with a haem–T1Cu distance of ∼10 Å is rate limiting in the reduction of the T1Cu site in native *Rp*NiR. Based on the observation that the axial ligand mutation (Met144Leu) significantly elevates the T1Cu reduction potential in *Ax*NiR and other T1Cu-containing proteins, the equivalent Met148Leu mutation was introduced in *Rp*NiR. This was done to investigate whether an increased T1Cu potential would reduce the activity, as the tethering of the haem domain (the fused physiological donor) should remove the intermolecular electron transfer as the rate-determining factor in catalysis. Indeed, against the background in wt*Rp*NiR, the Met148 Leu variant showed a 40% decrease at pH 5.5 (and 70% decrease at pH 6.5) in activity when ascorbate/PMS was used as reductant. This is further evidence in support of the hypothesis that in the two-component encounter complex, the formation of the complex and subsequent intermolecular electron transfer between the physiological donor and T1Cu centre of the enzyme is the rate-determining factor of enzyme turnover.

**Figure 4:**
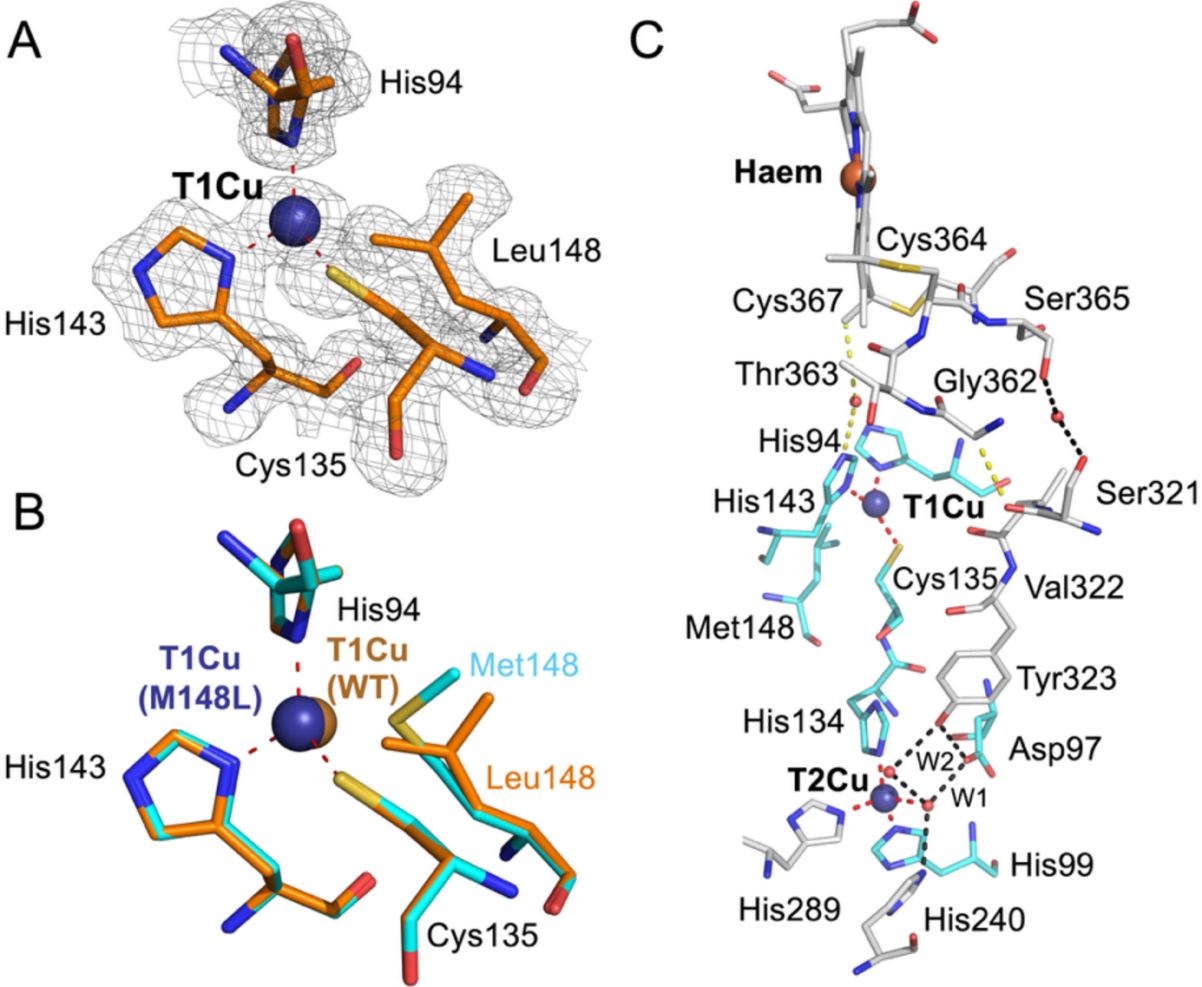
Comparison of the T1Cu copper site in wt*Rp*NiR and Met148Leu *Rp*NiR, and its electron transfer route. (A) Details of the T1Cu site of the Met148Leu *Rp*NiR structure and (B) a superposition of wt*Rp*NiR (3ziy) and Met148Leu. *Rp*NiR shows the movement of T1Cu in Met148Leu *Rp*NiR towards the His143 due to the loss of the axial ligand upon mutation. (C) Electron transfer route of Met148Leu from the haem to T1Cu, and T2Cu. The Met148Leu is shown in orange, and the wild type is shown in blue. The 2*F*_o_ − *F*_c_ electron-density map is shown as a grey mesh at the 1σ level. T1Cu is shown as a deep blue sphere and a brown sphere for Met148Leu and wild type, respectively. The red dashed lines represent coordination bonds of the copper sites.

The structure of the *Rp*NiR variant Met148Leu does not change significantly compared with wt*Rp*NiR with a C^α^ atom r.m.s. displacement for 453 atoms of 0.1 Å. As expected, differences are observed at the T1Cu site, while the ET route from the haem to the core domain is similar to that of the wild-type enzyme (Fig. 4 ▸). The distance from T1Cu to C^δ1^Leu148 in the Met148Leu variant (2.96 Å) is longer than the S^γ^Met148 distance in the wt*Rp*NiR (2.58 Å) (Fig. 4 ▸ and Table S2), indicating a weaker interaction. The T1Cu has moved into the plane created by S^γ^Cys135, N^δ1^His94 and N^δ1^His143, and adopts a trigonal-planar geometry with C^δ1^Leu148 2.96 Å away from the Cu ion, which is similar to the position of Leu144 in Met144Leu *Ax*NiR (Hough *et al.*, 2005 ▸). The T2Cu conformation and arrangement of coordinating residues are unchanged (Table S2). These observations suggest that lower activity is a consequence of a slower rate of inter-Cu ET due to an increase in the reorganizational energy of the T1Cu centre arising from the change in geometry (Farver *et al.*, 2004 ▸) and a weaker driving force for ET from T1Cu to T2Cu.

### 3.5. Structures of the as-isolated and NO-soaked Gln262Asn variant of *Rp*NiR highlight modulation of the primary proton channel

**Figure 5:**
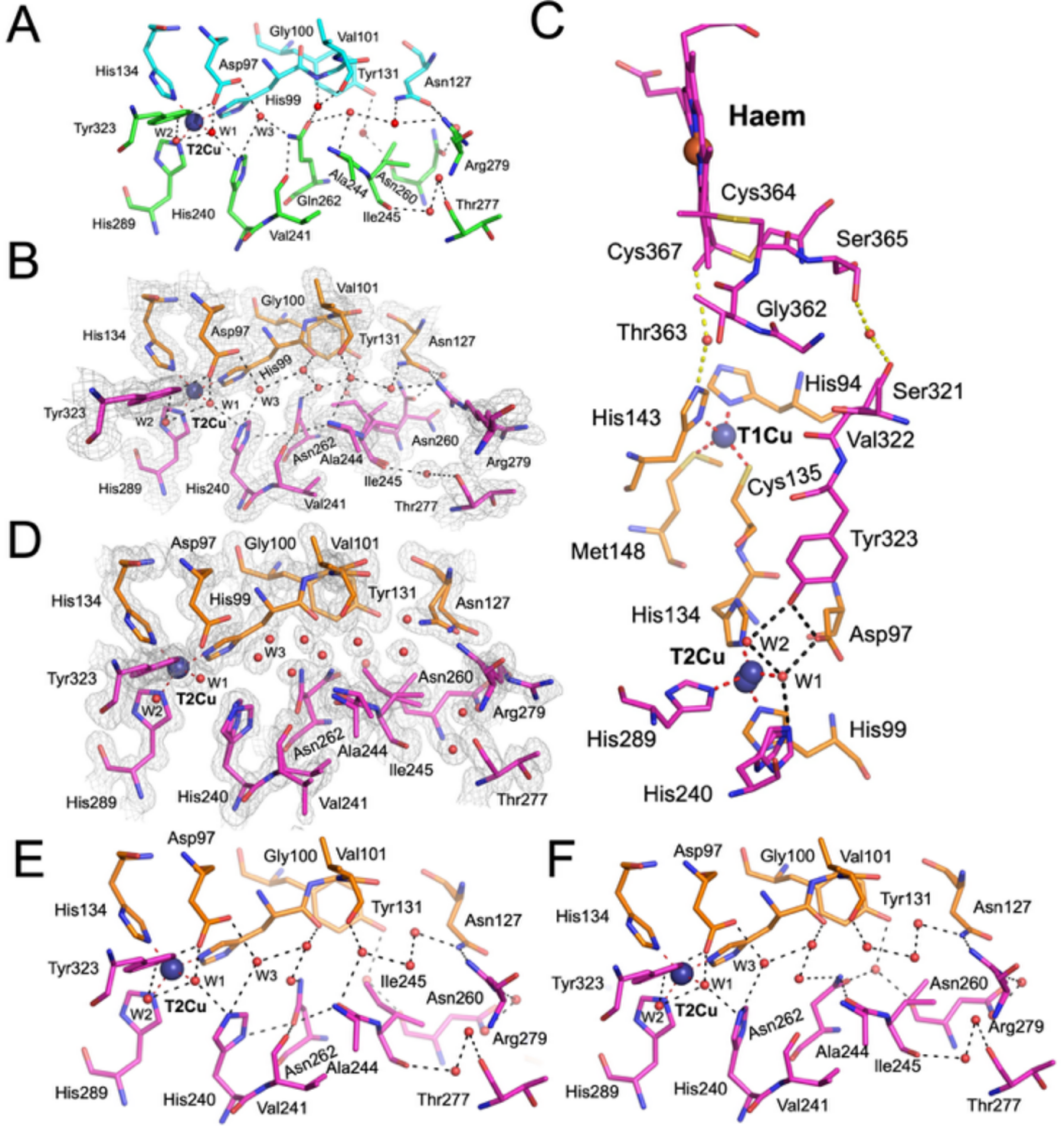
Conformation of residues in the primary proton channel and T2Cu site of wt*Rp*NiR, as-isolated and NO-soaked Gln262Asn *Rp*NiR mutant structures. (A) wt*Rp*NiR channel. (B) The water-filled channel in as-isolated Gln262Asn *Rp*NiR. (C) Electron transfer route of NO-soaked Gln262Asn from the haem to T1Cu, and T2Cu; (D) In NO-soaked Gln262Asn *Rp*NiR, two states of Gln262Asn can be observed. The details of the interaction of residues in state 1 are shown in (E) and in state 2 in (F). For simplicity, Tyr131 is only shown when it is involved in the water-network-related interactions. Small red spheres represent water molecules, the black dashed lines are the hydrogen bonds, the red dashed lines are the interactions involving T2Cu and the deep blue sphere is T2Cu. Gln262Asn *Rp*NiR is shown in magenta and orange. The 2*F*_o_ − *F*_c_ electron-density map is contoured at the 1σ level and shown as a grey mesh.

In prototypic two-domain CuNiRs, two putative proton channels linking the T2Cu centre to the enzyme surface have been identified. One has been established as a hydro­phobic substrate access channel (secondary proton channel) by mutational studies (Ellis *et al.*, 2002 ▸). Access to the primary proton channel is blocked by a conserved His254 residue (Hough *et al.*, 2008 ▸; Ellis *et al.*, 2002 ▸). In the haem-tethered CuNiRs, the primary channel is not obstructed (Antonyuk *et al.*, 2013 ▸) and has been shown to adopt two alternative states (Hedison *et al.*, 2019 ▸). The orientation of a conserved residue Ile245 (Ile235 in *Ph*NiR and His254 in *Ax*NiR) is proposed to control solvent accessibility to the active site pocket [Fig. 5 ▸(A), Fig. S3(A)]. The conformation of several residues lining the channel, notably the main chain of His99 and Gly100, are also different between *Ph*NiR (Tsuda *et al.*, 2013 ▸) and *Rp*NiR (Antonyuk *et al.*, 2013 ▸). Interestingly, residue Gln262 (*Rp*NiR), located ∼6 Å away from T2Cu, adopts two different conformations – an upward conformation in wt*Rp*NiR and downward conformation in *Ph*NiR, and also in the genetically engineered core of *Rp*NiR (Hedison *et al.*, 2019 ▸) (Fig. S3). In the wt*Rp*NiR (open primary proton state), Gln262 is in the upward conformation and connected with Asp_CAT_*via* W3, the main-chain nitro­gen of Gly100, and the main-chain oxygen of Val241 *via* another water [Fig. 5 ▸(A)]. W3 interacts with Asp97 and weakly with His240. On the other hand, Gln252 in *Ph*NiR is in the downward conformation (closed primary proton state) and its side-chain oxygen atom is directly bonded to the main-chain atoms of Ile122, Tyr121 and N^δ1^His89 (Fig. S3).

Given that the conformations and interactions of Gln262 may be important for modulating the primary proton states in three-domain cyt *c* tethered CuNiRs, we constructed the Gln262Asn variant of *Rp*NiR to test this hypothesis. As noted in Fig. S1, this variant shows a threefold increase in activity at pH 6.5 compared with wt*Rp*NiR. The crystal structure was solved in the space group *H*3 with a resolution of 1.5 Å [Fig. 5 ▸(B)]. The overall structure is similar to the wt*Rp*NiR, with the gatekeeper Tyr323 residue remaining in the locked-down position (r.m.s. deviation of C^α^ atoms 0.1 Å). Although there are numerous conformational changes of residues in the primary proton channel, the water channel is not disrupted. The conformation of Asn262 is in an upward position and it is turned towards catalytic His240 [Fig. 5 ▸(B) and Table S3]. The atom O^δ1^ of Asn262 interacts with the main-chain carbonyl of Ala244 and N^δ1^ of catalytic His240, which is stabilized by W1 and a weak interaction with W3. The N^δ1^ of Asn262 is stabilized by a water molecule that connects to the water network and main-chain oxygen of His99. The presence of Asn262 results in flexibility of Ile245, which is proposed to be the primary proton channel activator. Another characteristic of Gln262Asn *Rp*NiR is the flipping of the main-chain O of His99 and the main-chain N of Gly100 changing its configuration to that seen in *Ph*NiR, and in the isolated core of *Rp*NiR (Fig. S3).

Soaking of the crystals of Gln262Asn *Rp*NiR with NO allowed us to determine additional states of the primary proton channel [Fig. 5 ▸(D, E, F)]. This structure was also solved in the space group *H*3 at an atomic resolution of 1.09 Å, allowing the use of unrestrained *SHELXL* refinement. Two states of the primary proton channel are observed. In state 1, the O^δ1^ of Asn262 interacts with the main-chain O of Val241, Ala244 and N^δ1^ of the catalytic His240 [Fig. 5 ▸(E)]. The imidazole ring of the catalytic His240 rotates away from the T2Cu to a conformation observed in *Ph*NiR [Fig. S2(A)], where it retains the interaction with W1 (the distance N^ɛ2^His240 to W1 is 2.8 Å). W3 is present and moves closer to the N^δ1^ of Asn262. The flexibility of Ile245 is also observed, as seen in the as-isolated structure. In state 2, the side-chain O^δ1^ of Asn262 is stabilized by the interaction with a water molecule and the main-chain O of Ala244. The N^δ1^ of Asn262 connects to Tyr131 *via* a water molecule [Fig. 5 ▸(F)]. The catalytic His240 adopts the conformation seen in wt*Rp*NiR.

The unrestrained *SHELXL* refinement allowed a detailed analysis of the bond lengths and angles of catalytic Asp97 and His240 in the 1.09 Å resolution structure of NO-soaked Gln262Asn *Rp*NiR. The protonation state of Asp_CAT_ is negatively charged [O^δ1^—C^γ^ is 1.266 (1) Å and O^δ2^—C^γ^ is 1.252 (5) Å], like that of reduced wt*Rp*NiR (Fig. S6, Table S1). The distances between N^ɛ2^ of His_CAT_and W1 in the open and closed states are slightly different (2.9 and 2.8 Å, respectively). The C—N—C angles associated with the N^δ1^ and N^δ2^ atoms of His_CAT_ show that the protonation states in state 1 and 2 are different. In state 1, His_CAT_ is positively charged [C^γ^—N^δ1^—C^ɛ1^ is 109.6 (3.4)° and C^ɛ1^—N^ɛ2^—C^δ2^ is 108.2 (4.0)°], while in state 2 His_CAT_ is neutral where N^δ1^ is protonated and N^δ2^ is deprotonated [C^γ^—N^δ1^—C^ɛ1^ is 107.8 (1.1)° and C^ɛ1^—N^ɛ2^—C^δ2^ is 106.1 (4.2)° (Table S1)]. The differences in protonation states likely result from the presence of NO. While several changes in the primary proton channel are observed upon mutation of Gln262, the increase in enzymatic activity reinforces its role as a modulator of the primary proton channel. The hydrogen atoms observed in the hydrogen omit map for the T2Cu site and the haem are shown in Figs. S7 and S9, respectively, indicating the high quality of the structures.

### 3.6. Structures of as-isolated, NO-soaked and di­thio­nite-reduced activity-enhanced Phe295Leu *Rp*NiR – increased accessibility of hydro­phobic cavity and weakened Asp97 and Tyr323 interaction

**Figure 6:**
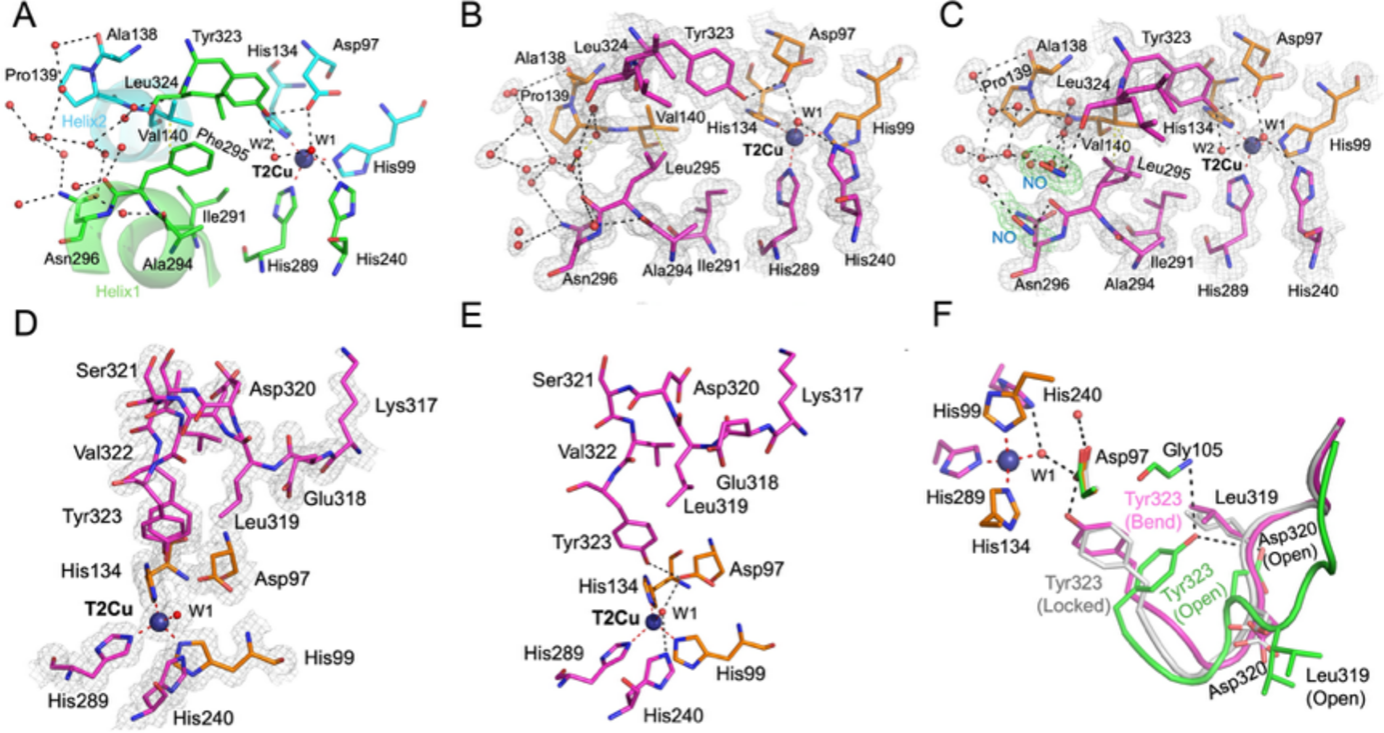
Conformation of residues in the hydro­phobic proton cavity and T2Cu site of as-isolated, NO-soaked Phe295Leu, and the T2Cu site of Phe295Leu reduced *Rp*NiR mutant structures. The interaction of residues at the entrance cavity is shown in (A) wt*Rp*NiR (3ziy), (B) as-isolated Phe295Leu *Rp*NiR with electron-density map and details of interactions, and (C) NO-soaked Phe295Leu *Rp*NiR with electron-density map and details of interactions, revealing two alternative conformations of Leu295. The yellow dotted line indicates the closest distance between residues Phe295 and Val140. (D) Di­thio­nite-soaked Phe295Leu *Rp*NiR with electron-density map. (E) Details of Tyr323 interactions in the bent conformation along with linker loop residues 317–322. (F) Alignment of three Tyr323 conformations, including locked down (3ziy), open (5ocf) and bent conformation, are shown in grey, green and magenta sticks, respectively. The 2*F*_o_ − *F*_c_ electron-density map is contoured at 1.0σ and shown as a grey mesh for as-isolated and NO-soaked *Rp*NiR Phe295Leu structures and as a blue mesh for di­thio­nite-soaked Phe295Leu *Rp*NiR. Phe295Leu *Rp*NiR is shown in magenta and orange, and wt*Rp*NiR in green and blue. Small red spheres represent water molecules, the black dashed lines are the hydrogen bonds, the red dashed lines are the interactions involving T2Cu and the deep blue sphere is T2Cu.

The hydro­phobic proton cavity of *Rp*NiR is partially buried by the cyt *c* domain and located close to the water network at the interface between the cyt *c* and the core enzyme, ∼7 Å away from the T2Cu site. The access to this channel is limited by the hydro­phobic interaction of Phe295 and Val140 [Fig. 6 ▸(A)]. The modification of the equivalent residue Phe306 in *Ax*NiR and Phe312 in *Af*NiR at the entrance of the hydro­phobic channel to the T2Cu has been shown to allow greater accessibility of substrate/small molecules in two-domain CuNiRs (Leferink *et al.*, 2014 ▸; MacPherson *et al.*, 2010 ▸; Adman *et al.*, 1995 ▸). In *Ax*NiR, substitution of Phe with Cys altered the water structure at the T2Cu site, weakened the apparent *K*_m_ for nitrite and resulted in a fourfold increase in specific activity due to a change in the rate-limiting step (Farver *et al.*, 2004 ▸). Based on the mutational study on prototypic CuNiRs, we investigated the effect of improving access to the cavity in *Rp*NiR by constructing the Phe295Leu substitution. The mutation results in a two- or fourfold increase in activity depending on the pH (Fig. 2 ▸, Fig. S1).

The structures of as-isolated and NO-soaked Phe295Leu were both solved in the space group *H*3 at 1.16 Å resolution. The substitution resulted in small changes in the global structure compared with wt*Rp*NiR, with the r.m.s. deviation between C^α^ atoms of 0.05 Å and 0.09 Å, respectively. In the as-isolated Phe295Leu *Rp*NiR, more space is available above Leu295, which adopts a single conformation in which C^δ1^ is positioned ∼3.3 Å from C^β^ of Val140 [Fig. 6 ▸(B)]. The conformation of the nearby residue Ile291, which belongs to the helix (residues 291–297) protecting the T2Cu site, is affected and is different from that of wt*Rp*NiR, correlating with the conformation of Leu295. This conformation of Ile291 has been seen previously in the structures of the *Ph*NiR (Tsuda *et al.*, 2013 ▸) and the *Rp*NiR core (Antonyuk *et al.*, 2013 ▸) (Figs. S4 and S5). The conformational change of Ile291 is triggered since the phenyl ring of Phe295 does not restrain its side chain. The T2Cu water molecule, W2, which usually interacts with Tyr323 is absent, presumably allowing effective substrate binding, suggesting that the absence of W2 may represent the activated state of Tyr323. Interestingly, analysis of bond lengths and angles of catalytic residues of as-isolated Phe295Leu, allowed by unrestrained *SHELXL* refinement, is consistent with Asp97_CAT_ being deprotonated and His240_CAT_ has its N^δ1^ protonated and N^ɛ2^ non-protonated, as seen in reduced wt*Rp*NiR (Tables S1 and S7).

In the NO-soaked Phe295Leu structure, Leu295 adopts two conformations [Fig. 6 ▸(C) and Fig. S6]. The additional conformation of Leu295, with its C^δ1^ and C^δ2^ turned away from C^β^ of Val140, may be a consequence of the presence of NO, one near the main-chain O atom of Leu324 and the second close to the side chain of Asn296. The conformation of the nearby residue Ile291 is the same as in as-isolated Phe295Leu *Rp*NiR. The water molecule W2 re-appears in the NO-soaked structure. The comparison of the water network between wt*Rp*NiR, activated Tyr323, NO-bound wt*Rp*NiR and as-isolated Phe295Leu *Rp*NiR shows that the water network connecting Ala138 O to Tyr323 O, and Asn296 O to Ala294 O, is intact [Fig. 6 ▸(A–C) and Fig. S6]. The water network is disturbed by NO in the NO-soaked Phe295Leu *Rp*NiR. Analysis of the bond lengths and angles of catalytic Asp97 and His240 in as-isolated and NO-soaked Phe295Leu *Rp*NiR is enabled by unrestrained *SHELXL* refinement. The protonation state of Asp97_CAT_ observed in the reduced enzyme is unchanged on NO soaking of crystals. This residue is negatively charged [O^δ1^—C^γ^ is 1.255 (1) Å and O^δ2^—C^γ^ is 1.265 (3) Å for as-isolated Phe295Leu, and O^δ1^—C^γ^ is 1.253 (7) Å and O^δ2^—C^γ^ is 1.268 (5) Å for NO-soaked Phe295Leu], as seen in reduced wt*Rp*NiR. The C—N—C angles associated with the N^δ1^ and N^δ2^ atoms of His_CAT_ show that the reduced protonation states in both Phe295Leu *Rp*NiR structures are similar, with N^δ1^ protonated and N^δ2^ deprotonated [C^γ^—N^δ1^—C^ɛ1^ is 109 (1)°, C^ɛ1^—N^ɛ2^—C^δ2^ is 106 (1)° for as-isolated Phe295Leu and C^γ^—N^δ1^—C^ɛ1^ is 107 (1)°, C^ɛ1^—N^ɛ2^—C^δ2^ is 104 (1)° for NO-soaked Phe295Leu] (Table S1). The protonation states of Asp97_CAT_ and His240_CAT_ in as-isolated and NO-soaked Phe295Leu suggest that the catalytic state is primed for binding nitrite, since W2 is no longer present and the interactions between Asp97 and Tyr323 are weakened. Moreover, the accessibility of the hydro­phobic cavity is enhanced, which all together could explain the substantially increased activity for this mutant. However, despite various soaking attempts, we were unable to obtain the nitrite-bound structure of the Phe295Leu variant. An important unanswered question that remains is what triggers the return of the W2 molecule upon NO soaking and if it is linked to the inability to successfully form a stable nitrite-bound species.

The structure of di­thio­nite-reduced Phe295Leu *Rp*NiR was also solved in the space group *H*3 at 1.33 Å resolution [Fig. 6 ▸(D)]. Changes were observed at both the T1Cu and T2Cu sites. The T1Cu site showed two conformations of Met148, like that seen in reduced wt*Rp*NiR [Fig. 1 ▸(B), Fig. S4(B)]. The T2Cu site retained W1 but lacked water molecule W2. Strikingly, the linker loop containing Tyr323 of reduced Phe295Leu *Rp*NiR adopts an alternative conformation, which we term the ‘bent’ conformation (the locked-down conformation occupancy is 0.75, and the occupancy of the bent conformation is 0.25) [Fig. 6 ▸(D)]. In this conformation, the OH group of Tyr323 can interact with O^δ1^ of Asp97 (the distance is ∼2.7 Å, compared with the locked-down conformation of ∼2.5 Å) but its aromatic ring is bent [Fig. 6 ▸(E)]. The main-chain conformations of residues Leu322 and Tyr323 are poised to flip along with a modest shift of residues Ser321 and Asp320. This results in more space above Tyr323, which might create a cavity for substrate/small molecules/water [Fig. 6 ▸(F)]. This new conformation of Tyr323 suggests that it may become more mobile (or activated) on reduction of the T2Cu centre (Dong *et al.*, 2018 ▸). The ‘bent’ conformation of Tyr323 could potentially be the transition state between the locked-down and the activated state. This is further reinforced by the increased activity observed for this mutant.

## 4. Conclusion

The discovery of three-domain copper nitrite reductases over 15 years ago (Ellis *et al.*, 2007 ▸; Nojiri *et al.*, 2007 ▸) and structural characterization of a few C-terminal cytochrome (Antonyuk *et al.*, 2013 ▸; Tsuda *et al.*, 2013 ▸) or cupredoxin domains revealed three redox centres [T1Cu_C_–T1Cu_N_–T2Cu in *Thermus scotoductus* SA-01, *Ts*NiR (Opperman *et al.*, 2019 ▸) and CytFe–T1Cu_N_–T2Cu in *Rp*NiR and *Ph*NiR] well poised for efficient ET to the T1Cu centre of the core enzyme. Despite this seemingly obvious advantage, all of these self-contained systems exhibit significant lower enzymatic activity than the prototypic two-domain CuNiRs when receiving electrons from their cognate redox partner(s). This has led some to question whether the tethered domain is functionally relevant as the redox donor, since structure of the three-domain enzyme from *Hyphomicrobium denitrificans* placed the T1Cu centre in the tethered domain too far away from the T1Cu centre of the core enzyme (Nojiri *et al.*, 2007 ▸).

*Rp*NiR is the best-studied tethered CuNiR, but even in this case it has not been possible to obtain a substrate-bound enzyme structure of the wild-type enzyme, and as such it has been difficult to establish the nature of fundamental processes that underpin nitrite catalysis in this system. Here, we present the first structure of reduced *Rp*NiR and have combined structure–function studies of several mutants of the enzyme that exhibit activity spanning over a fourfold range with Phe295Leu and Met148Leu at the extremes.

**Figure 7:**
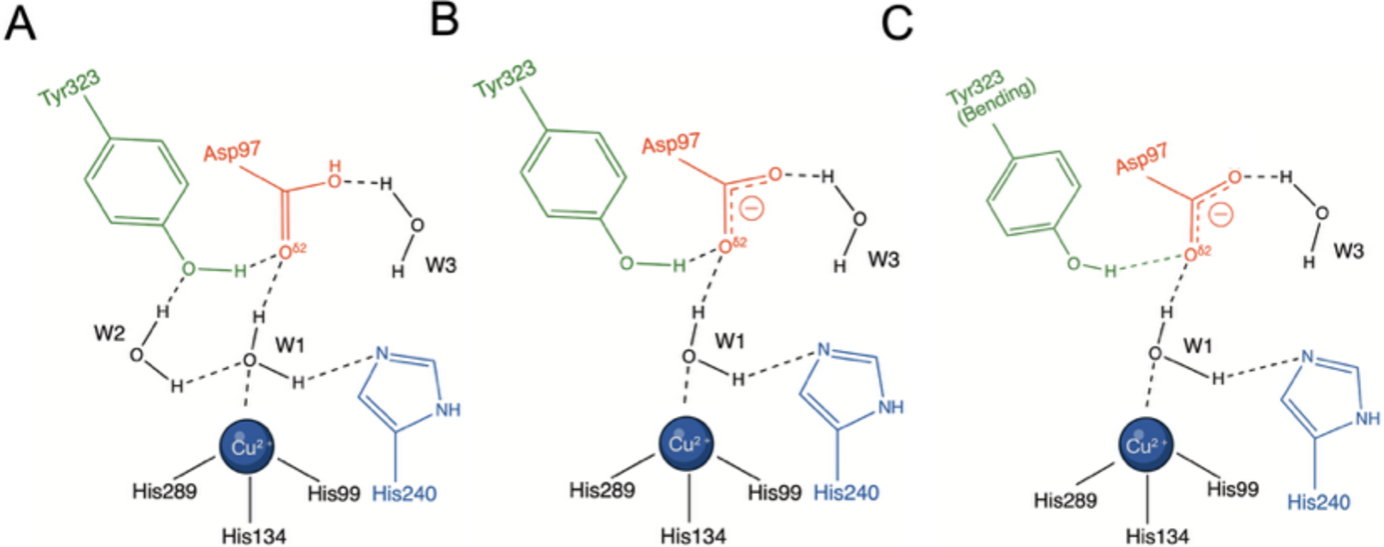
Illustration of protonation states of catalytic Asp97 and His240. (A) The resting state of catalytic T2Cu site in wt*Rp*NiR (3ziy) shows the presence of W2 and doubly hydrogen-bonded O^δ2^Asp97 to W1 and Tyr323. (B) The reduced state of catalytic T2Cu site in wt*Rp*NiR shows that W2 is lost and Asp97 is negatively charged. (C) The reduced state of catalytic T2Cu site in Phe295Leu *Rp*NiR shows the bent conformation of Tyr323, and similar states as in reduced wt*Rp*NiR.

Although we have kept the X-ray dose to a level (Table S5) where the T1Cu in CuNiR remains largely oxidized (Rose *et al.* 2024 ▸) to determine an atomic resolution structure, it is significantly higher than where the haem centre can be transitioned to a ferrous state (Pfanzagl *et al.*, 2020 ▸). Our atomic resolution (<1.2 Å) structure of di­thio­nite-reduced *Rp*NiR shows the changes that occur in the catalytic pocket and in the second coordination sphere of the T2Cu, and enabled unrestrained *SHELXL* refinement to assign probable protonation states of the Asp_CAT_ and His_CAT_ residues. The substrate access channel of oxidized *Rp*NiR has Tyr323 in the locked-down position, which is stabilized by the interaction of the Asp_CAT_ side chain and the water molecule W2 [Fig. 7 ▸(A)]. On reduction of the T2Cu site, W2 is lost and the protonation state of Asp_CAT_ changes, becoming negatively charged. Together these changes weaken the anchoring of Tyr323 in the catalytic channel to allow substrate access [Fig. 7 ▸(B)]. The available nitrite-bound structures of variants of *Rp*NiR show nitrite binds by displacement of both water ligands, consistent with recent XFEL atomic resolution structures of two-domain CuNiRs from various species (Rose *et al.*, 2026 ▸). We also observed a new bent conformation of Tyr323 in the di­thio­nite-reduced Phe295Leu *Rp*NiR structure [Fig. 7 ▸(C)]. In this conformation, the OH side chain of Tyr323 still interacts with Asp_CAT_, but it results in increased flexibility of residues 320–323 in the linker loop. This suggests that the conformational change of Tyr323 and the linker loop following reduction may be the trigger for the open conformation, allowing substrate to reach the catalytic site.

The structure of *Rp*NiR has a water-mediated network at the interface between the tethered and core domains with a distance between the haem edge to T1Cu of ∼10.1 Å. It has been shown that mutations of Met92 and Pro93, which directly contact the haem, have no significant effect on the specific activity of the enzyme (Antonyuk *et al.*, 2013 ▸), supporting a water-mediated pathway for ET. The presence of additional ET routes from the haem to the catalytic pocket (haem c > Cys364 > Gly362 > **Ser321** > Tyr323, and haem c > Cys364 > Ser365 > water > **Ser321** > Tyr323) is supported by Ser321Met *Rp*NiR with significant increase in the specific activity. The effect on activity suggests that ET in *Rp*NiR can be improved by introducing hydro­phobic through-bond interactions in the water-mediated network.

One benefit of the tethering domain may be the protection of *Rp*NiR from reductive inactivation in the absence of substrate, since inter-Cu ET cannot occur until nitrite binds to the oxidized T2Cu centre. The mechanism that relays the oxidation state of the haem to allow nitrite to bind to the T2Cu is unclear. It has been shown that the conformation of Tyr323 is unaltered in the T2D-*Rp*NiR (type-2 depleted Cu enzyme) and as such is not affected by the absence of the T2Cu centre (Dong *et al.*, 2018 ▸). Our structure of reduced *Rp*NiR provides some insight, since we observe flexibility of Gly362–Thr363 located in the cyt *c* domain, and this subtle conformational change results in the alternate conformation of the T1Cu ligand Met148 (distal conformation), which increases the Cu—S^γ^ bond length and may provide the signal for regulating the electron transfer between two domains.

We have also identified Gln262 as a key residue, modulating the water network in the primary proton channel. Mutation of Gln262 causes multifaceted changes in the channel, such as a conformational change of the channel activator, Ile245, and flipping of the main chain of His99–Gly100, features that are also seen in the resting-state structure of *Ph*NiR. In the NO-soaked Gln262Asn structure, although NO cannot be seen bound in the structure, two additional features can be observed: flexibility of Asn262 and a double conformation of catalytic His240. One of the conformations (state 2) of His240 is like that of *Ph*NiR, in which it is unprotonated and therefore unable to function as a proton donor in catalysis. Given that this inert His240 conformation is present in the presence of NO, the primary proton channel may also be involved in the product release. The doubling of activity for the Phe295Leu mutant and the observation of NO molecules close to residue 295, namely the main-chain O atom of Leu324, and the side chain of Asn296 suggest that this is the likely route for substrate/product channelling to the bulk solvent.

Given the recent emergence of *AlphaFold3* (Jumper *et al.*, 2021 ▸), to accommodate multimeric proteins we generated the structures of the variants using the online *AlphaFold3* server. A comparison with experimental structures gave mixed results. Predicted conformations of mutated residues in Gln262Asn and Ser321Met were different from the experimental structures, while the conformation of Leu148 in the Met148Leu variant is very similar in the predicted and experimental structures. Interestingly, in Phe295Leu, it showed that Tyr323 has lost the bond to Asp_CAT_ rather than a weaker interaction between Tyr323 and Asp_CAT_. These are consistent and support our observation that the Phe295Leu mutation provides the conformational flexibility of the linker loop, influencing the conformation of Tyr323 and activating it with resulting higher activity.

## Supplementary Material

Supporting tables and figures. DOI: 10.1107/S2052252526004549/lz5078sup1.pdf

PDB reference: as-isolated Ser321Met mutant of *Rp*NiR, 7qq2

PDB reference: as-isolated Met148Leu mutant of *Rp*NiR, 8qgf

PDB reference: as-isolated Gln262Asn mutant of *Rp*NiR, 7r2u

PDB reference: as-isolated Phe295Leu mutant of *Rp*NiR, 9fom

PDB reference: reduced Phe295Leu mutant of *Rp*NiR, 9fuj

PDB reference: reduced wt*Rp*NiR, 9fuh

PDB reference: NO-treated Gln262Asn mutant of *Rp*NiR, 9fuk

PDB reference: NO-treated Phe295Leu mutant of *Rp*NiR, 9fui

## Figures and Tables

**Table 1:** Data collection and refinement statistics of as-isolated *Rp*NiR mutants Values for the highest-resolution shell are shown in parentheses.

	Ser321Met *Rp*NiR (as-isolated)	Met148Leu *Rp*NiR (as-isolated)	Gln262Asn *Rp*NiR (as-isolated)	Phe295Leu *Rp*NiR (as-isolated)
Data collection				
PDB ID	7qq2	8qgf	7r2u	9fom
Space group	*I*2_1_3	*H*3	*H*3	*H*3
Wavelength (Å)	0.91260	0.97856	0.99990	0.80000
Cell dimensions				
*a*, *b*, *c* (Å)	180.14, 180.14, 180.14	127.95, 127.95, 86.25	127.65, 127.65, 86.29	128.28, 128.28, 86.19
α, β, γ (°)	90, 90, 90	90, 90, 120	90, 90, 120	90, 90, 120
Resolution (Å)	73.65–2.1 (2.16–2.1)	46.66–1.32 (1.34–1.32)	37.63–1.50 (1.53–1.50)	32.08–1.16 (1.19–1.16)
No. of reflections	56514	124091	83956	182213
*R*_merge_	0.114 (1.011)	0.137 (1.888)	0.108 (1.324)	0.088 (0.993)
CC_1/2_	0.996 (0.546)	0.995 (0.265)	0.996 (0.431)	0.996 (0.433)
〈*I*/σ(*I*)〉	8.1 (1.5)	6.4 (0.7)	8.3 (1.2)	5.8 (1.39)
Completeness (%)	99.9 (100)	99.4 (87.9)	100 (100)	99.5 (99.8)
Redundancy	4.6 (4.7)	5.1 (4.5)	5.0 (5.0)	3.6 (3.5)
Wilson *B* factor (Å^2^)	29.7	13.4	15.3	12.5
				
Refinement				
*R*_work_/*R*_free_	0.153/0.186	0.134/0.162	0.108/0.153	0.114/0.139
No. of atoms				
Protein	3437	3738	3439	3790
Ligand/ion	43/2	43/2	43/2	43/2
Water	429	770	613	822
*B* factors (Å^2^)				
Protein	34.35	16.83	24.35	15.81
Ligand/Cu	41.22/29.85	14.30/13.20	15.45/16.34	12.65/11.62
Water	43.04	33.17	43.62	34.90
R.m.s. deviations				
Bond lengths (Å)	0.013	0.012	0.018	0.011
Bond angles (°)	1.93	1.68	2.05	1.69

**Table 2:** Data collection and refinement statistics for chemically reduced and NO-soaked *Rp*NiR mutants Values for the highest-resolution shell are shown in parentheses.

	Reduced Phe295Leu *Rp*NiR (di­thio­nite-soaked)	Reduced wt*Rp*NiR (di­thio­nite-soaked)	Gln262Asn *Rp*NiR (NO-soaked)	Phe295Leu *Rp*NiR (NO-soaked)
Data collection				
PDB ID	9fuj	9fuh	9fuk	9fui
Space group	*H*3	*H*3	*H*3	*H*3
Wavelength (Å)	0.80001	0.87000	0.97625	0.86999
Cell dimensions				
*a*, *b*, *c* (Å)	127.98, 127.98, 86.58	127.85, 127.85, 86.35	128.07, 128.07, 86.35	128.28, 128.28, 86.35
α, β, γ (°)	90, 90, 120	90, 90, 120	90, 90, 120	90, 90, 120
Resolution (Å)	37.71–1.33 (1.35–1.33)	37.68–1.17 (1.19–1.17)	37.71–1.09 (1.11–1.09)	37.03–1.17 (1.19–1.17)
No. of reflections	116526	177553	205295	173732
*R*_merge_	0.085 (1.072)	0.089 (1.642)	0.046 (0.907)	0.063 (0.853)
*R*_p.i.m._	0.045 (0.754)	0.044 (0.836)	0.03 (0.684)	0.032 (0.588)
CC_1/2_	0.998 (0.363)	0.998 (0.354)	0.999 (0.344)	0.999 (0.504)
〈*I*/σ(*I*)〉	8.0 (0.8)	7.9 (0.9)	11.1 (1.3)	10.3 (1.32)
Completeness (%)	96.1 (71.8)	100 (100)	87.2 (82.9)	97.2 (73.2)
Redundancy	4.3 (2.5)	4.9 (4.8)	2.5 (1.9)	4.7 (2.8)
Wilson *B* factor (Å^2^)	13.8	12.2	11.8	12.0
				
Refinement				
*R*_work_/*R*_free_	0.112/0.141	0.122/0.146	0.107/0.122	0.106/0.128
No. of atoms				
Protein	3829	3639	3821	3834
Ligand/ion	43/2	43/2	43/3	43/2
Water	742	824	880	850
*B* factors (Å^2^)				
Protein	18.51	18.87	12.53	13.71
Ligand/Cu	17.49/13.54	16.94/14.07	9.33/9.37	12.09/10.36
Water	36.79	38.11	30.8	30.65
R.m.s. deviations				
Bond lengths (Å)	0.012	0.014	0.010	0.011
Bond angles (°)	1.86	1.88	1.77	1.80
