# Supplementary material for: Functional role of a tethered domain as a naturally fused cognate partner is demonstrated in a three-domain copper nitrite reductase
Source: IUCrJ. 2026 Jun 15;13(Pt 4):498–513. doi: 10.1107/S2052252526004549 (PMC13324648; doi:10.1107/S2052252526004549)
Supplement: Supplementary file 1 [file m-13-00498-sup1.pdf]

IUCrJ

Volume 13 (2026)

Supporting information for article:

Functional role of a tethered domain as a naturally fused cognate partner is demonstrated in a three-domain copper nitrite reductase

Nopphon Petchyam, Allegra Mbouku, Robert Eady, Samar Hasnain and Svetlana Antonyuk

Table S1. Bond lengths and angles of catalytic Asp97 and His240 of *Rp*NiR variants by unrestrained SHELXL refinement

Distances (Å) and Angles (°)	**As-isolated wt*Rp*NiR**	**Reduced wt*Rp*NiR**	**As-isolated F295L *Rp*NiR**	**NO soaked F295L *Rp*NiR**	**NO soaked Q262N *Rp*NiR**
**Resolution (Å)**	**1.0**	**1.17**	**1.16**	**1.17**	**1.09**
**PDB**	**3ZIY**	**9FUH**	**9FOM**	**9FUI**	**9FUK**
**Asp97**					
Occupancy	1.00	1.00	1.00	1.00	1.00
C^{γ}-$O^{\delta 1}$ (Å)	1.282(9)	1.259(9)	1.255(1)	1.268(5)	1.266(1)
C^{γ}-$O^{\delta 2}$ (Å)	1.242(8)	1.270(9)	1.265(3)	1.253(7)	1.252(5)
$N\sigma^{\#}$	3.33	0.86	3.16	1.74	2.75
**His240**					
Occupancy	1.00	1.00	1.00	1.00	0.6 (open state)/ 0.4 (close state)
$C^{\varepsilon 1}$-$N^{\varepsilon 2}$-$C^{\delta 2}$ (°)	105(1)	105(2)	106(1)	104(1)	106(4)/108(4)*
C^{γ} -$N^{\delta 1}$-$C^{\varepsilon 1}$ (°)	109(1)	108(2)	109(1)	107(1)	108(1)/110(3)*
$N\sigma^{\#}$	2.83	1.06	2.12	2.12	0.485/0.4

*Bond lengths and angles of His240 in Q262N NO soaked *Rp*NiR are shown for open and close states respectively.
e.s.d.s values to the last digit in brackets

#Z-score ($N\sigma$) for the Asp97, and His240 is calculated from the following equation $N\sigma = \frac{|\Delta|}{\sqrt{e.s.d.s1^2 + e.s.d.s2^2}}$
where Δ is the difference between bond length of C^{γ}-$O^{\delta 1}$ and Cγ-Oδ2 for Asp97, and the bond angle of Cε1-Nε2-Cδ2 and Cγ -Nδ1-Cε1 for His240.

Table S2. Distances between T1Cu and T2Cu to their ligands of all *Rp*NiR mutants (Å)

	As-isolated wildtype	Reduced wildtype	As-isolated M148L

	As-isolated F295L	Dithionite soaked F295L	NO soaked F295L

PDB ID	3ZIY	9FUH	8QGF
T1Cu			
His94 $N^{\delta1}$	2.02	2.01	2.02
Cys135 S^{γ}	2.23	2.21	2.19
His143 $N^{\delta1}$	2.06	2.05	2.03
Met148 S^{δ}	2.58	2.67/ 4.40 (proximal/ distal)	2.96 (Leu148$C^{\delta1}$)
T2Cu			
His134 $N^{\varepsilon2}$	2.00	2.00	2.00
His289 $N^{\varepsilon2}$	2.00	1.99	2.01
His99 $N^{\varepsilon2}$	2.00	2.03	1.99
W1	2.07	2.05	2.06
Presence of W2	Yes	No	Yes

	As-isolated S321M	As-isolated Q262N	NO soaked Q262N
PDB ID	7QQ2	7R2U	9FUK
T1Cu			
His94 $N^{\delta1}$	2.16	2.03	2.02
Cys135 S^{γ}	2.21	2.22	2.21
His143 $N^{\delta1}$	2.04	2.04	2.05
Met148 S^{δ}	2.57	2.55	2.56
T2Cu			
His134 $N^{\varepsilon2}$	2.01	2.04	1.96
His289 $N^{\varepsilon2}$	2.02	2.03	2.04
His99 $N^{\varepsilon2}$	1.93	1.98	1.86
W1	2.03	2.16	2.15
Presence of W2	No	Yes	Yes

T1Cu			
His94 $N^{\delta 1}$	2.04	1.98	2.02
Cys135 S^{γ}	2.22	2.21	2.21
His143 $N^{\delta 1}$	2.10	2.09	2.08
Met148 S^{δ}	2.52	2.62/4.38 (proximal/ distal)	2.54
T2Cu			
His134 $N^{\varepsilon 2}$	1.98	2.01	2.04
His289 $N^{\varepsilon 2}$	1.99	2.00	2.04
His99 $N^{\varepsilon 2}$	2.05	2.00	2.02
W1	2.03	2.06	1.99
Presence of W2	No	No	Yes

Table S2. (contd.) Distances between T1Cu and T2Cu to their ligands of all *Rp*NiR mutants (Å)

Table S3. Summary of the primary proton channel states in *Rp*NiR structures

Structural features	wt*Rp*NiR (3ZIY)	wt*Ph*NiR (2ZOO)	As-isolated Q262N *Rp*NiR	NO soaked Q262N *Rp*NiR
Primary proton state defined by the conformations of Ile245 (Ile)	Open	Close	Open and close	Open and close
Conformation of Gln262 (Gln in *Ph*NiR)	Upward	Downward	Upward	Upward
Double conformations of His240	-	-	-	State 1 is similar to that of *Ph*NiR and state 2 is similar to wt*Rp*NiR
Conformation of mainchain O His99 and N of Gly100	Not flipped	Flipped	Flipped	Flipped

Table S4. Bond lengths and angles of haem c of *Rp*NiR variants by unrestrained SHELXL refinement

Distances (Å) and Angles (°)	**As-isolated wt*Rp*NiR**	**Reduced wt*Rp*NiR**	**As-isolated F295L *Rp*NiR**	**NO soaked F295L *Rp*NiR**	**NO soaked Q262N *Rp*NiR**
**Resolution**	**1.0 Å**	**1.17 Å**	**1.16 Å**	**1.17 Å**	**1.09 Å**
**PDB**	**3ZIY**	**9FUH**	**9FOM**	**9FUI**	**9FUK**
**Haem c**					
Occupancy	1.00	1.00	1.00	1.00	1.00
Fe-NA (Å)	1.989(9)	1.990(7)	1.980(7)	1.972(8)	1.971(3)
Fe-NB (Å)	1.999(3)	1.995(8)	2.003(7)	1.979(9)	1.995(5)
Fe-NC (Å)	1.971(6)	1.990(8)	1.975(9)	1.980(4)	1.951(8)
Fe-ND (Å)	1.994(9)	1.999(0)	2.008(4)	1.953(7)	2.017(5)
Fe-$N^{\varepsilon2}$ of His368 (Å)	1.976(9)	2.014(0)	2.010(4)	1.953(7)	1.982(9)
Fe-S^{δ} of Met418 (Å)	2.327(8)	2.334(8)	2.329(1)	2.334(5)	2.331(5)
NA-Fe-NB (°)	90.5 (0.3)	90.9 (0.9)	90.4 (0.5)	88.5 (0.5)	90.5 (0.3)
NA-Fe-NC (°)	179.6 (0.4)	177.9 (1.0)	179.2 (0.6)	179.3 (0.6)	178.7 (0.4)
NA-Fe-ND (°)	89.0 (0.3)	89.0 (0.9)	89.2 (0.5)	91.6 (0.5)	88.0 (0.3)
NA-Fe-$N^{\varepsilon2}$ of His368 (°)	91.2 (0.3)	93.5 (0.9)	92.4 (0.5)	91.8 (0.5)	92.0 (0.3)
NA-Fe-S^{δ} of Met418 (°)	85.5 (0.2)	84.9 (0.7)	85.7 (0.4)	85.7 (0.4)	84.8 (0.3)
NB-Fe-NC (°)	89.9 (0.3)	89.4 (0.9)	89.5 (0.5)	88.5 (0.5)	90.4 (0.3)
NB-Fe-ND (°)	179.5 (0.3)	179.8 (1.4)	179.0 (0.6)	179.7 (0.7)	178.2 (0.3)
NB-Fe-$N^{\varepsilon2}$ of His368 (°)	88.8 (0.3)	88.2 (0.9)	87.4 (0.5)	88.6 (0.6)	87.8 (0.4)
NB-Fe-S^{δ} of Met418 (°)	92.0 (0.2)	91.9 (0.7)	92.6 (0.4)	91.6 (0.4)	93.0 (0.3)
NC-Fe-ND (°)	90.6 (0.3)	90.7 (0.9)	90.9 (0.5)	88.6 (0.5)	91.1 (0.3)

NC-Fe-$N^{\varepsilon2}$ of His368 (°)	88.8 (0.3)	88.5 (0.9)	88.4 (0.5)	88.7 (0.5)	89.0 (0.3)
NC-Fe-S^{δ} of Met418 (°)	94.4 (0.2)	93.1 (0.7)	93.5 (0.4)	93.7 (0.4)	94.1 (0.3)
ND-Fe-$N^{\varepsilon2}$ of His368 (°)	91.4 (0.3)	91.9 (0.9)	91.7 (0.6)	91.1 (0.5)	91.2 (0.4)
ND-Fe-S^{δ} of Met418 (°)	87.9 (0.2)	87.9 (0.7)	88.4 (0.4)	88.6 (0.4)	88.0 (0.3)
$N^{\varepsilon2}$ of His368-Fe-S^{δ} of Met418 (°)	176.7 (0.2)	178.4 (0.7)	178.1 (0.4)	177.5 (0.4)	176.7 (0.3)

* The errors in brackets are from unrestrained SHELXL refinement and relate to the last digit of the number. For medium-resolution structures, the bond errors are included in Table 1 of the main manuscript.

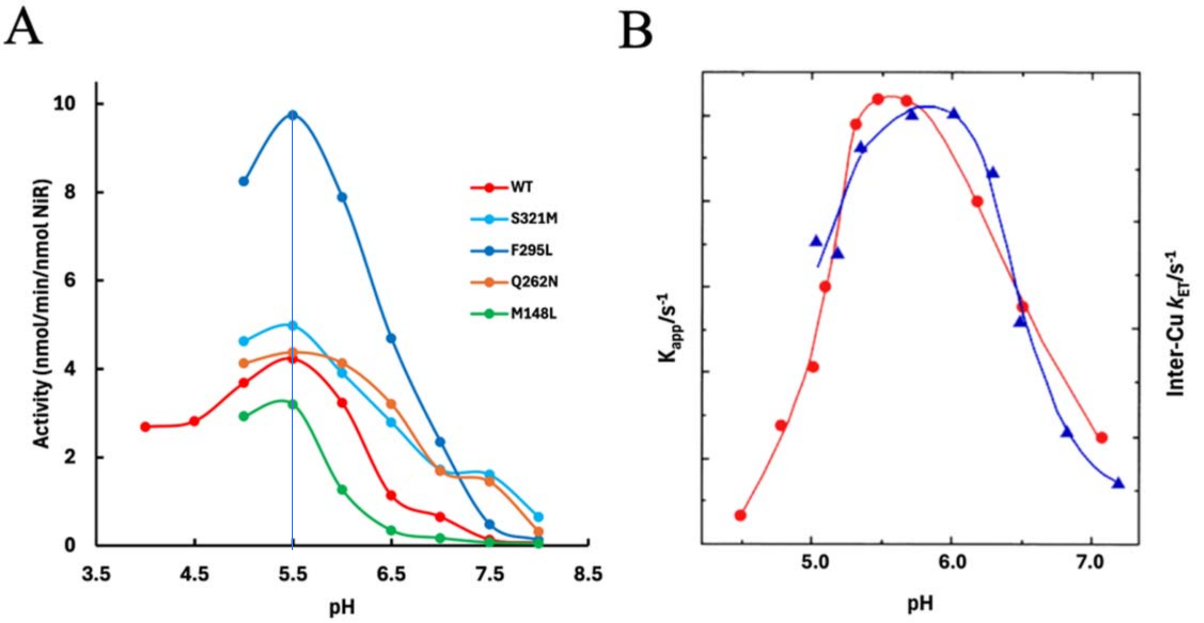

Figure S1. pH dependence of *Rp*NiR activity and comparison with prototypical *Ax*NiR. **A.** pH activity profiles of wt*Rp*NiR and mutants Ser321Met, Met148Leu, Phe295Leu, and Gln262Asn. All variants exhibit a similar bell-shaped pH dependence, with maximal activity at ~ pH 5.5. **B.** pH dependence of k_{CAT} and inter-Cu k_{ET} in the prototypical *Ax*NiR. The red curve represents the rate of inter-Cu electron transfer; the blue curve corresponds to k_{CAT}. The characteristic bell-shaped profile observed in prototypical CuNiRs arises from the protonation states of Asp_{CAT} and His_{CAT}. Figure adapted from Eady and Hasnain, Coordination Chemistry Reviews 460 (2022) 214463.

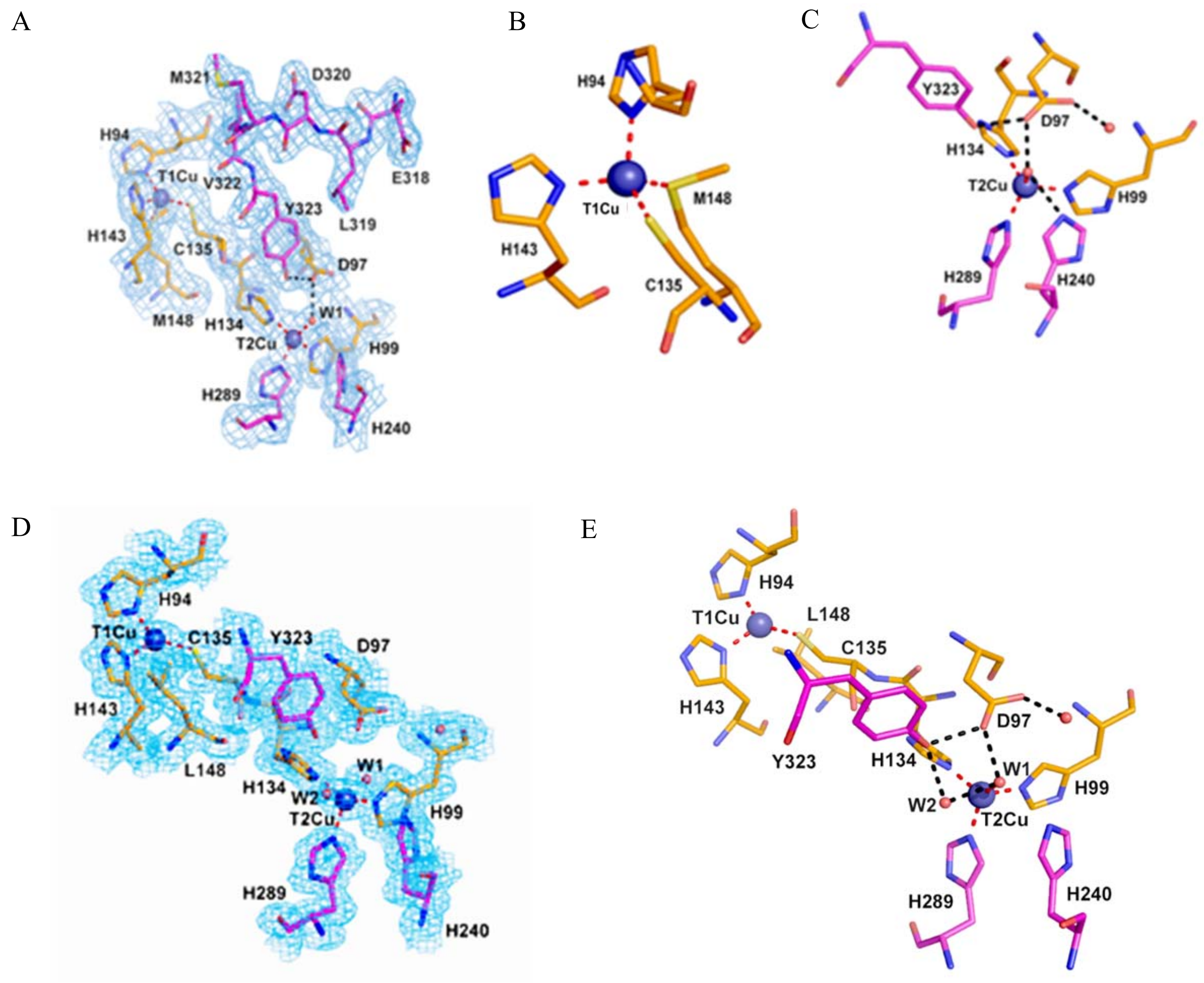

Figure S2. A close up view of T1Cu site and T2Cu sites of S321M *Rp*NiR. (A) 2Fo-Fc electron density map of Ser321Met *Rp*NiR contoured at 1σ level as blue mesh around Cu site residues and single water, W1. (B) T1Cu site with coordination bonds (C) T2Cu site with single W1 bound. (D) 2Fo-Fc electron density map of Met148Leu *Rp*NiR contoured at 1σ level as light blue mesh. (E) T1Cu and T2Cu site residues of as-isolated Met148Leu *Rp*NiR are shown as sticks. Two water molecules are located close to T2Cu, similar to wt*Rp*NiR. Small red spheres represent water molecules, the black dash lines are the hydrogen bonds, the red dash lines are the interactions involve in T2Cu, and the deep blue sphere is Cu ion. The residues from two neighbouring molecules participating in forming catalytic site; are shown in magenta and orange.

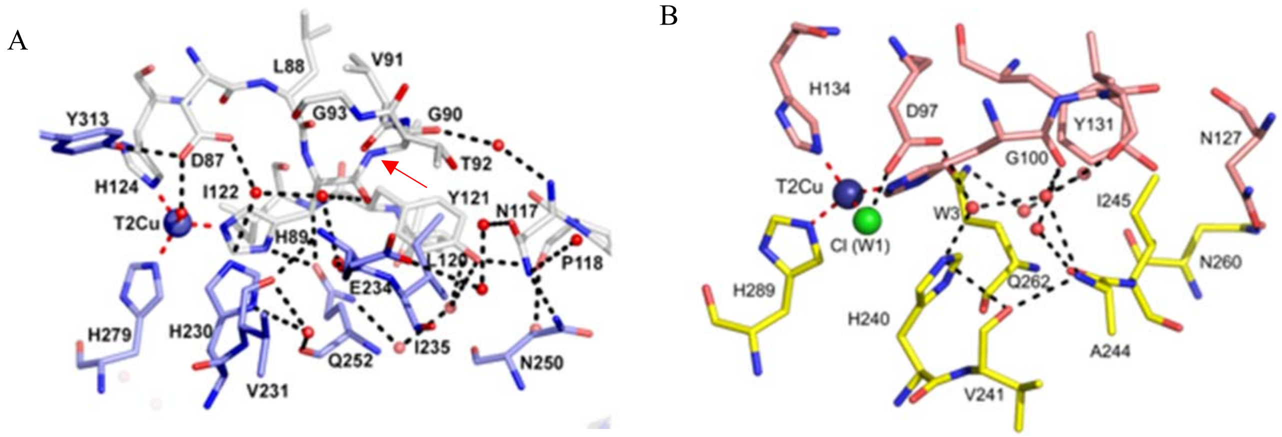

Figure S3. Conformation of residues in the primary proton channel and T2Cu site of *Ph*NiR, and truncated core of *Rp*NiR. (A) Proton channel of *Ph*NiR is closed due to the conformation of the sidechain of the Ile235 and Gln252 is in downward conformation. Residues of the neighbouring molecules are coloured in grey and slate, and red arrow indicates the flip of O His89 and N of Gly90 (B) Proton channel of the truncated core *Rp*NiR is in close state with residues coloured in pink and yellow, in which Ile245 is blocking the channel, and Gln262 is in downward conformation. Small red spheres represent water molecules, the black dash lines are the hydrogen bonds, the red dash lines areT2Cu coordinating bonds, and T2Cu is shown as the deep blue sphere. PDB code: 2ZOO for *Ph*NiR, and 6QPU for truncated core.

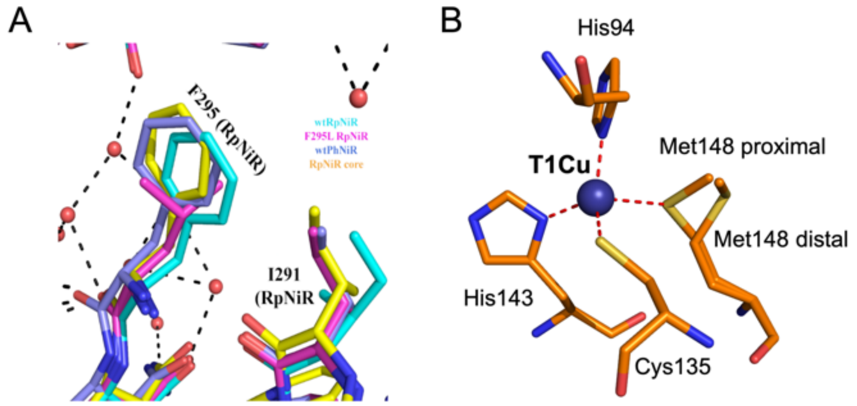

Figure S4. Alignment of three-domain cyt *c* tethered CuNiRs with the conformations of Phe295 and Ile291 and conformation of residues at T1Cu of reduced Phe295L structure. (A) The conformation of Phe295 and Ile291 (*Rp*NiR numbering) of wt*Rp*NiR (3ZIY) shown in blue, *Ph*NiR (2ZOO) shown in slate, truncated *Rp*NiR core (6QPU) shown in yellow, and as-isolated Phe295Leu *Rp*NiR shown in magenta is compared. Small red dots are water molecules, and hydrogen bonds are shown in black dotted lines. (B) T1Cu site of reduced Phe295L, Met148 adopts two conformations: with T1Cu to S^{δ} distances of 2.67 (proximal) and 4.4 (distal) Å.

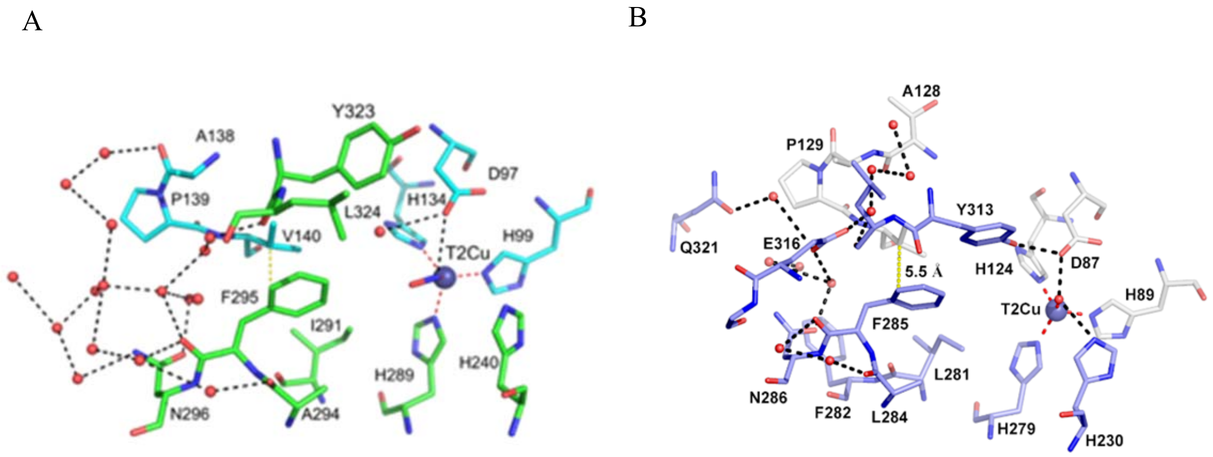

Figure S5. Comparison of the conformations of residues in hydrophobic proton cavity and T2Cu site of activated Tyr323-NO bound wt*Rp*NiR and *Ph*NiR. The interaction of residues at the entrance cavity is shown for (A) activated Tyr323-NO bound wt*Rp*NiR, (B) *Ph*NiR. Glu316 (*Ph*NiR numbering) occupies this space in *Ph*NiR due to arrangement of the linker. *Ph*NiR is shown in slate and grey, and wt*Rp*NiR is shown in blue and green. Small red spheres represent water molecules, the black dash lines are the hydrogen bonds, the red dash lines are the interactions involve in T2Cu, and the deep blue sphere is T2Cu. The yellow dotted line indicates the closet distance between residue 295 (285 in *Ph*NiR) and 140 (130 in *Ph*NiR).

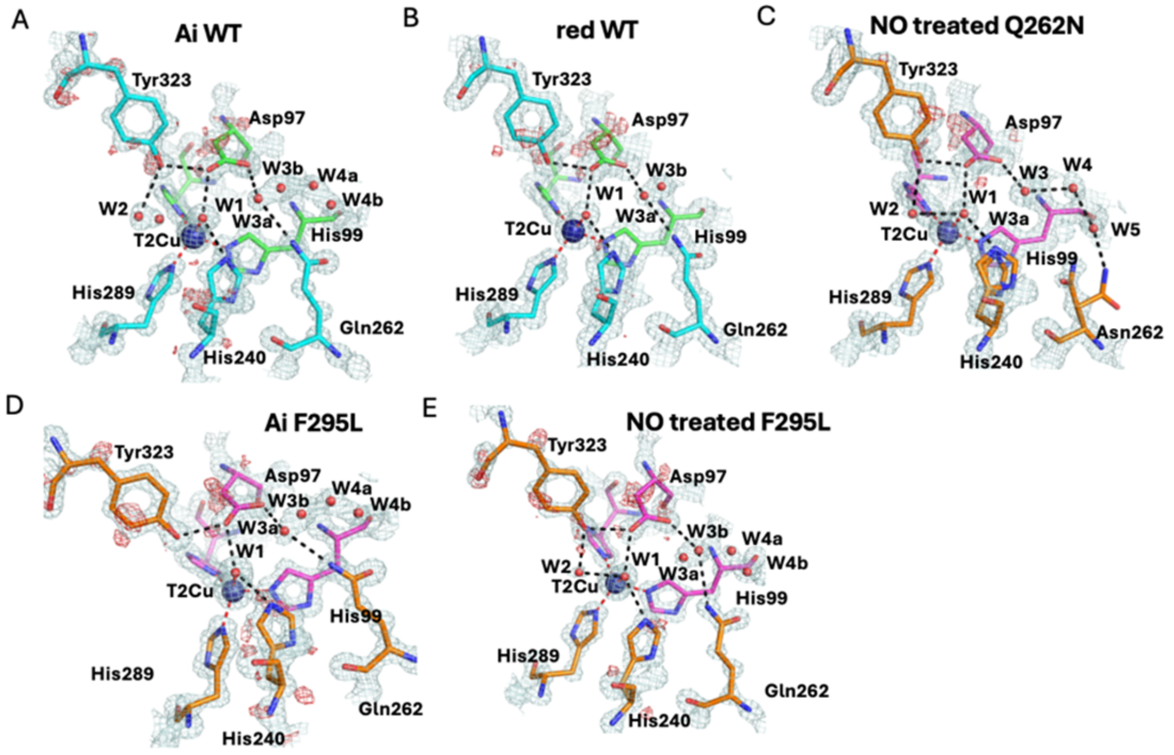

Figure S6. Active sites of the *Rp*NiR structures determined at atomic resolution. **A**. As isolated *Rp*NiR at 1.01 Å resolution, possible protonation of Asp97 and no protonation of His240 at $N^{\varepsilon 2}$; **B**. Reduced *Rp*NiR at 1.17 Å resolution, possible protonation of Asp97 and protonation of His240 at $N^{\varepsilon 2}$. **C**. NO treated Q262N *Rp*NiR mutant at 1.09Å resolution, showing a possible protonation of Asp97 and inconclusive protonation of His240 at $N^{\varepsilon 2}$ **D**. As isolated F295L *Rp*NiR mutant at 1.16 Å resolution, possible protonation of Asp97 and protonation of His240 at $N^{\varepsilon 2}$. **E.** NO treated as isolated F295L *Rp*NiR mutant at 1.17 Å resolution, showing a possible protonation of Asp97 and no protonation of His240 at $N^{\varepsilon 2}$. The residues are shown as sticks and coloured according to chains (wt protein in cyan and green; for mutants in magenta and orange), Cu ions are shown as dark blue spheres, and water molecules as small red spheres. $2F_o$-F_c electron-density map is contoured at 1.0σ and shown in grey mesh. Fo-Fc difference map is contoured at 2.5σ and coloured in red and may suggest the locations of the hydrogen atoms. For simplicity, the figure shows the omit maps for hydrogen atoms around Tyr323; Asp97 and His240.

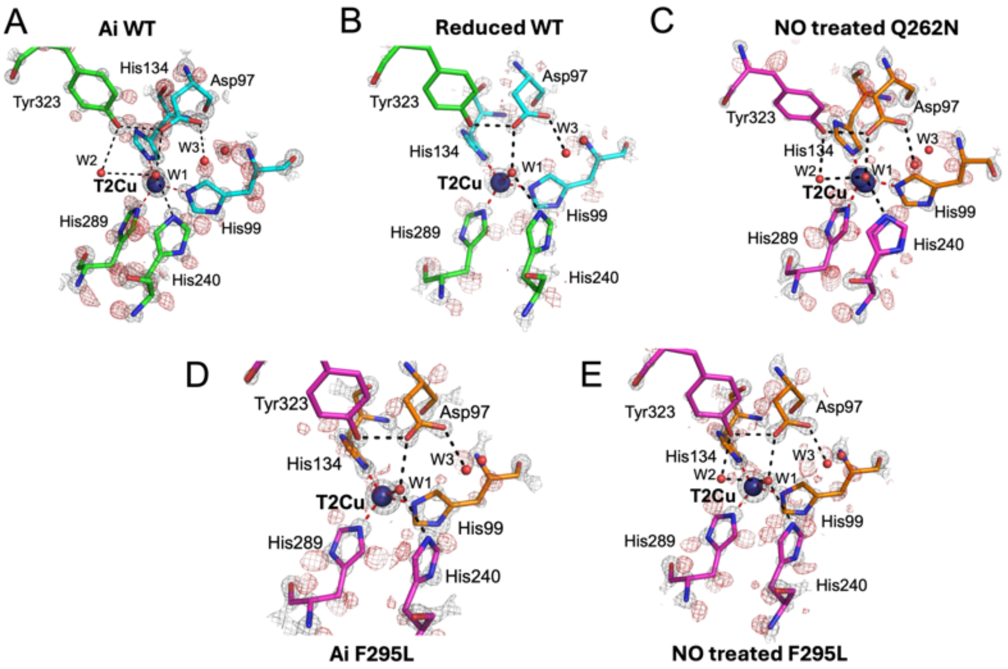

Figure S7. Omit maps around T2Cu site residues, indicating possible hydrogen positions. (A) as-isolated wt*Rp*NiR,(B) reduced wt*Rp*NiR, (C) NO soaked Gln262Asn, (D) as-isolated Phe295Leu, (E) NO soaked Phe295Leu *Rp*NiR mutants. 2mFo-DFc electron density map is shown as grey mesh at 5σ level, and Fo-Fc map is shown as red mesh at 2.5σ level. Small red spheres represent water molecules, the black dash lines are the hydrogen bonds, the red dash lines are the interactions involve in T2Cu, and the deep blue sphere is T2Cu. The reduced *Rp*NIR structure is shown in blue, and green, while the mutants are shown in magenta and orange.

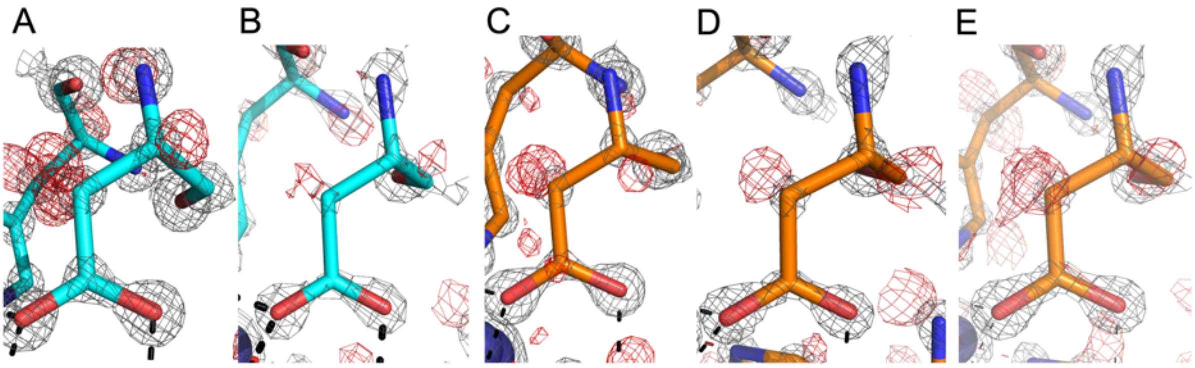

Figure S8. Asp97 of as-isolated wt*Rp*NiR (A), reduced wt*Rp*NiR (B), NO soaked Gln262Asn (C), as-isolated Phe295Leu (D), and NO soaked Phe295Leu (E) *Rp*NiR mutant structures with 2mFo-DFc electron density map shown at 5.0σ level, and Fo-Fc map at 2.5σ level. The reduced *Rp*NIR structure is shown in blue, and green, while the mutants are shown in magenta and orange. The 2mFo-DFc electron-density map is shown as grey mesh. The 2mFo-DFc electron-density map is shown as grey mesh.The Fo-Fc electron-density map is shown as red mesh.

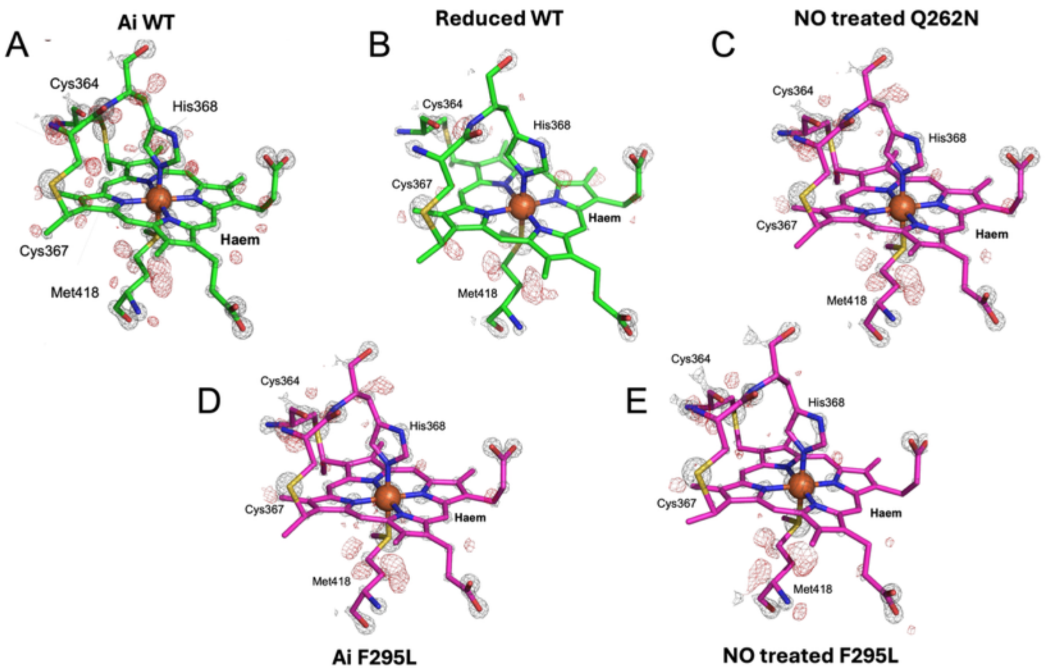

Figure S9. Omit maps around Haem C site, indicating possible hydrogen positions. as-isolated wt*Rp*NiR (A), reduced wt*Rp*NiR (B), NO soaked Gln262Asn (C), as-isolated Phe205Leu (D), and NO soaked Phe295Leu (E) *Rp*NiR mutants. 2mFo-DFc electron density map is shown as grey mesh at 5σ level, and Fo-Fc map is shown as red mesh at 2.5σ level. The reduced *Rp*NIR structure is shown in blue, and green, while the mutants are shown in magenta and orange.

Table S5. Radiation dose of *Rp*NiR structures.

*Rp*NiR structures	Radiation source	Wavelength (Å)	X-ray energy (keV)	Average radition dose (MGy)
S321M (as-isolated)	I04-01-DLS	0.91260	13.59	0.264
M148L (as-isolated)	Proxima-SOLEIL	0.97856	12.67	0.453
Q262N (as-isolated)	I24-DLS	0.99990	12.4	2.44
F295L (as-isolated)	I24-DLS	0.80000	15.50	1.49
Reduced F295L	I04-DLS	0.80001	15.49	0.181
Reduced wildtype	I24-DLS	0.87000	14.25	0.794
Q262N (NO soaked)	I03-DLS	0.97625	12.7	0.414
F295L (NO soaked)	I03-DLS	0.86999	14.25	1.35
